# Supplementary material for: Noncanonical amino acids as doubly bio-orthogonal handles for one-pot preparation of protein multiconjugates
Source: Nat Commun. 2023 Feb 21;14:974. doi: 10.1038/s41467-023-36658-y (PMC9944564; doi:10.1038/s41467-023-36658-y)
Supplement: Supplementary file 1 — Supplementary information [file 41467_2023_36658_MOESM1_ESM.pdf]

Supporting information

Title:

Noncanonical amino acids as doubly bio-orthogonal handles for one-pot preparation of protein multiconjugates

Authors: Yong Wang[1, 4], Jingming Zhang[2, 4], Boyang Han[1, 4], Linzhi Tan[1], Wenkang Cai[1], Yuxuan Li[1], Yeyu Su[1], Yutong Yu[1], Xin Wang[1], Xiaojiang Duan[2], Haoyu Wang[1], Xiaomeng Shi[1], Jing Wang[1], Xing Yang[2, 3*], Tao Liu[1*]

Affiliations

[1]State Key Laboratory of Natural and Biomimetic Drugs, Chemical Biology Center, Department of Molecular and Cellular Pharmacology, School of Pharmaceutical Sciences, Peking University 38 Xueyuan Road, Beijing 100191, China.

[2]Department of Nuclear Medicine, Peking University First Hospital, 100034 Beijing, China.

[3]Institute of Medical Technology, Peking University Health Science Center, 100191 Beijing, China.

[4]These authors contributed equally: Yong Wang, Jingming Zhang, Boyang Han.

*Correspondence to Xing Yang, Tao Liu.

✉Email: yangxing2017@bjmu.edu.cn; taoliupku@pku.edu.cn.

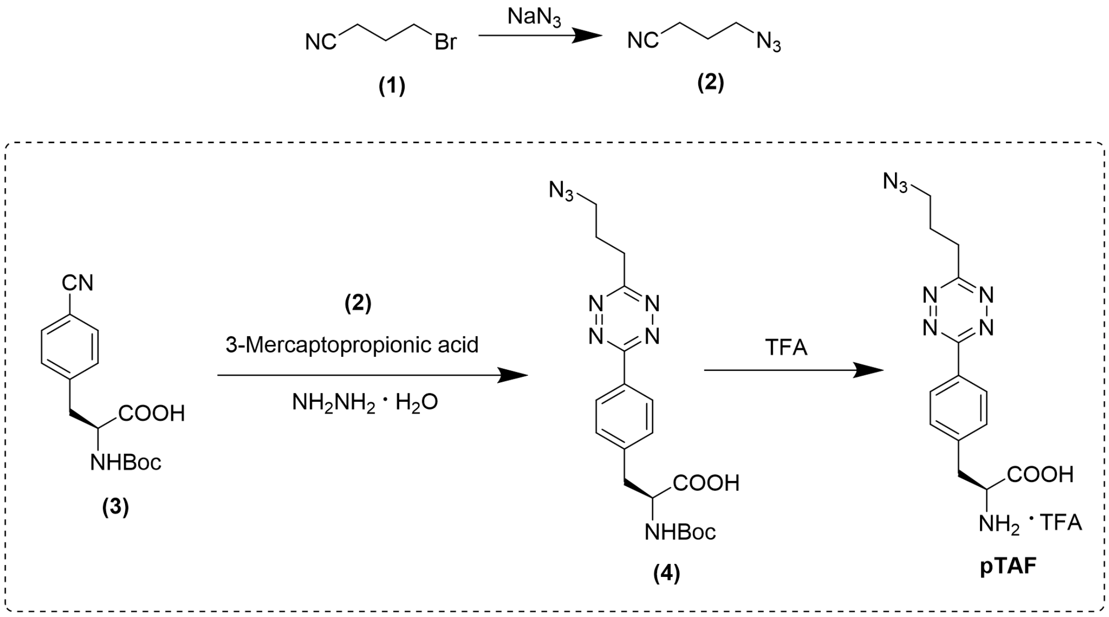

Supplementary Fig. 1. Synthetic route of pTAF. pTAF was synthesized by reaction of Boc-L-4-cyanophenylalanine (**3**) with 4-azidobutanenitrile (**2**) in the presence of hydrazine hydrate and 3-mercaptopropionic acid to form a 1,2-dihydrotetrazine, which was then oxidized and deprotected to give pTAF.

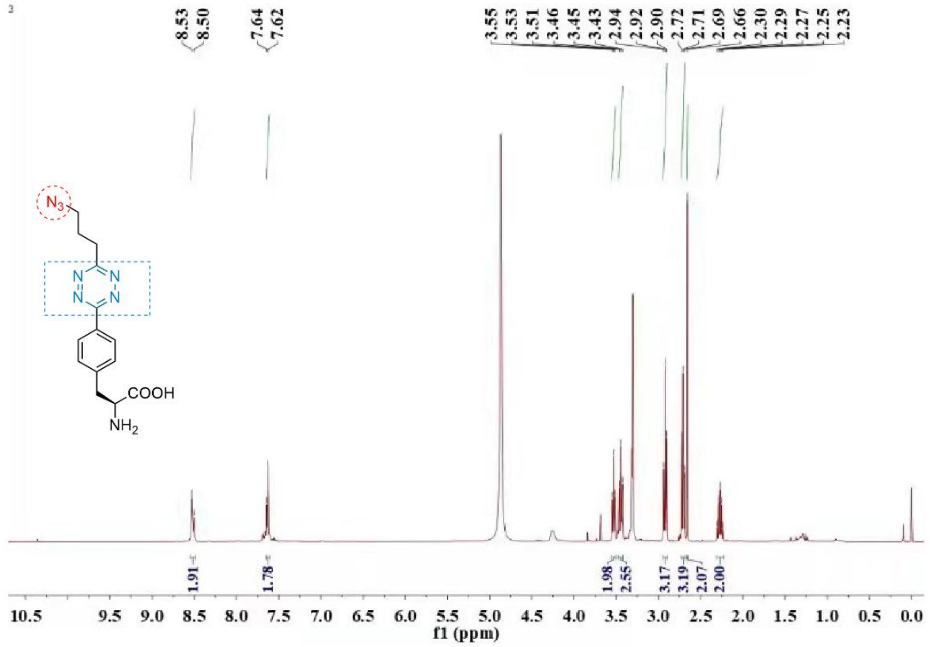

Supplementary Fig. 2. ^{1}H NMR spectra of pTAF

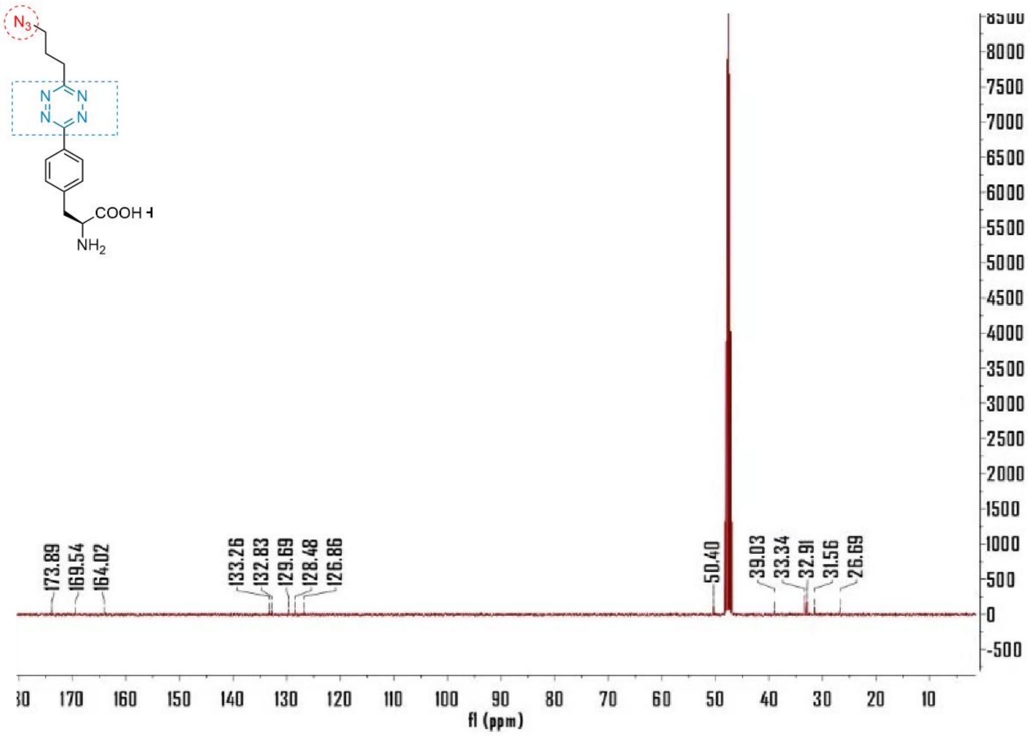

Supplementary Fig. 3. **^{13}C NMR spectra of pTAF.**

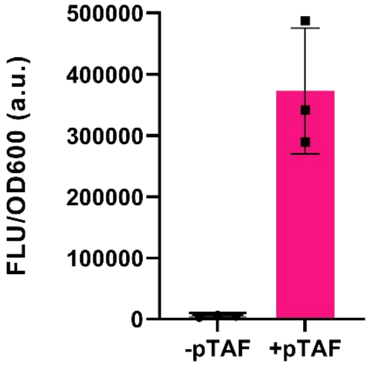

Supplementary Fig. 4. **A quantitative sfGFP fluorescence assay in the absence or presence of 1 mM pTAF.** SfGFP bearing amber codon was co-expressed with pTAFRS/tRNA pair containing plasmid. Samples were collected after 12 hours of cultivation. Fluorescence is shown after normalization to the OD at 600 nm in arbitrary units (a.u.). Data are presented as mean values ± s.d. (n = 3 biologically independent experiments). Source data are provided as a Source Data file.

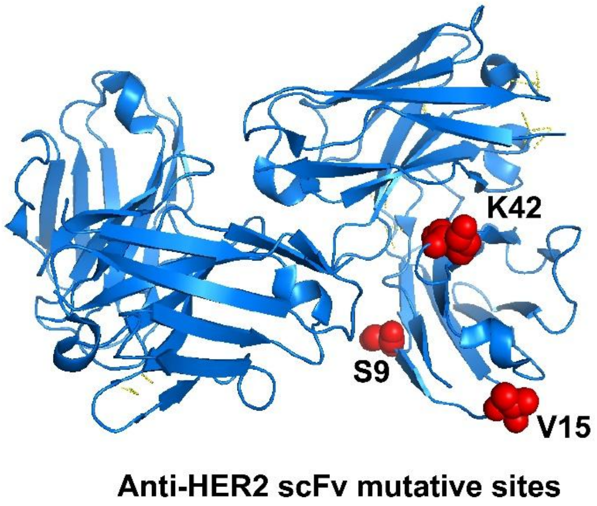

Supplementary Fig. 5. Screening the HER2-scFv region near the S9, K42, and V15 sites. The residues away from the binding site with high expression yield based the crystal structure of anti-HER2-scFv (PDB ID 6DN0).

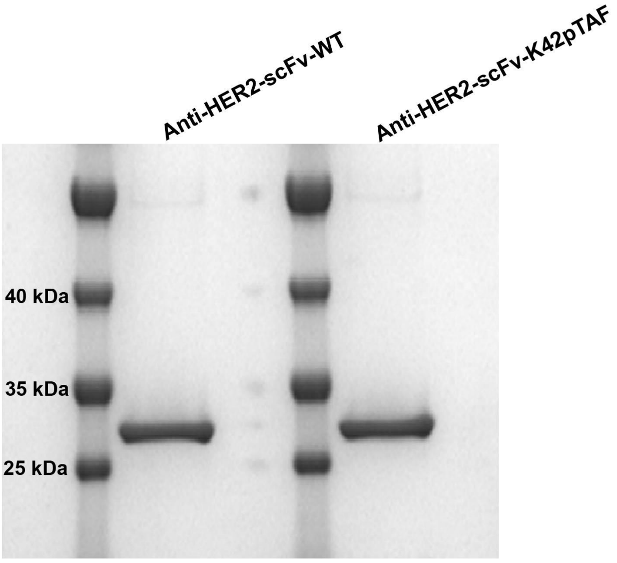

Supplementary Fig. 6. SDS-PAGE analysis of WT Anti-HER2-scFv and Anti-HER2-scFv-K42-pTAF mutant. Both proteins were purified from *E. coli* periplasmic space and migrated as a single band around 27 kDa. The purification experiments were repeated three times with reproducible results.

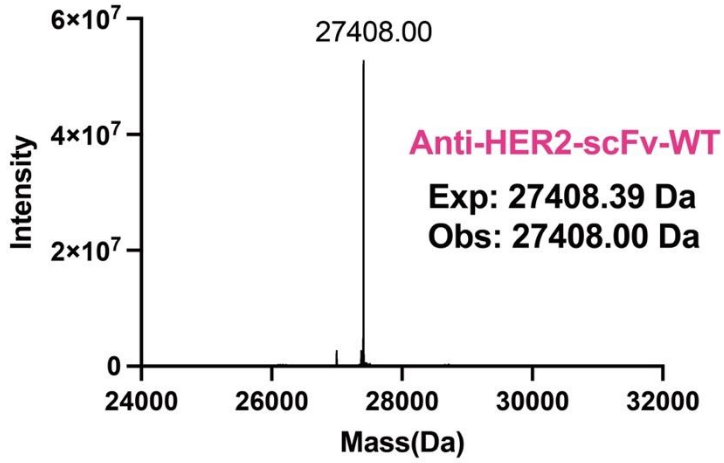

Supplementary Fig. 7. High-resolution electrospray-ionization mass spectrum of purified Anti-HER2-scFv-WT. Source data are provided as a Source Data file.

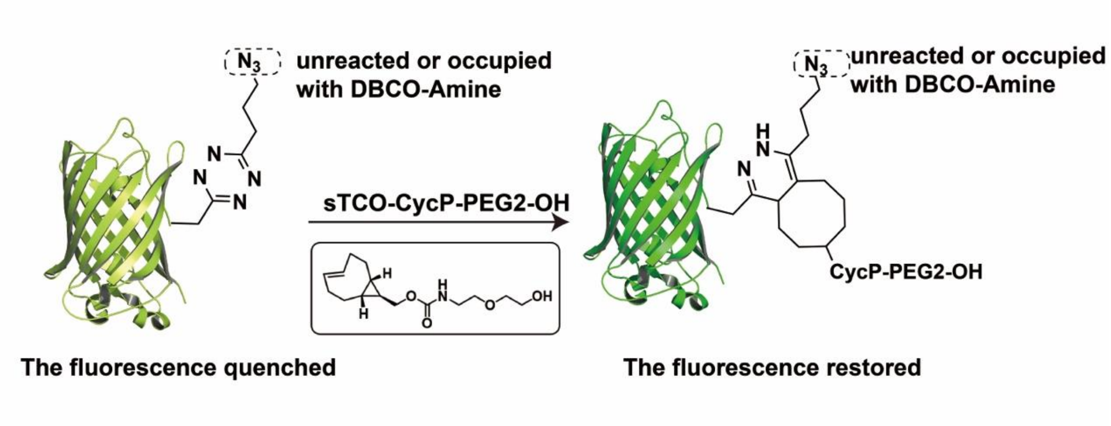

Supplementary Fig. 8. Determination of rate constants of sfGFP-Y151-pTAF with sTCO-CycP-PEG2-OH with azide unreacted or azide occupied. GFP fluorescence is quenched about 5 folds when pTAF is introduced at site Y151 with azide unreacted and azide occupied, but fluorescence returns with different degrees when reacted with sTCO-Cycp-PEG2-OH.

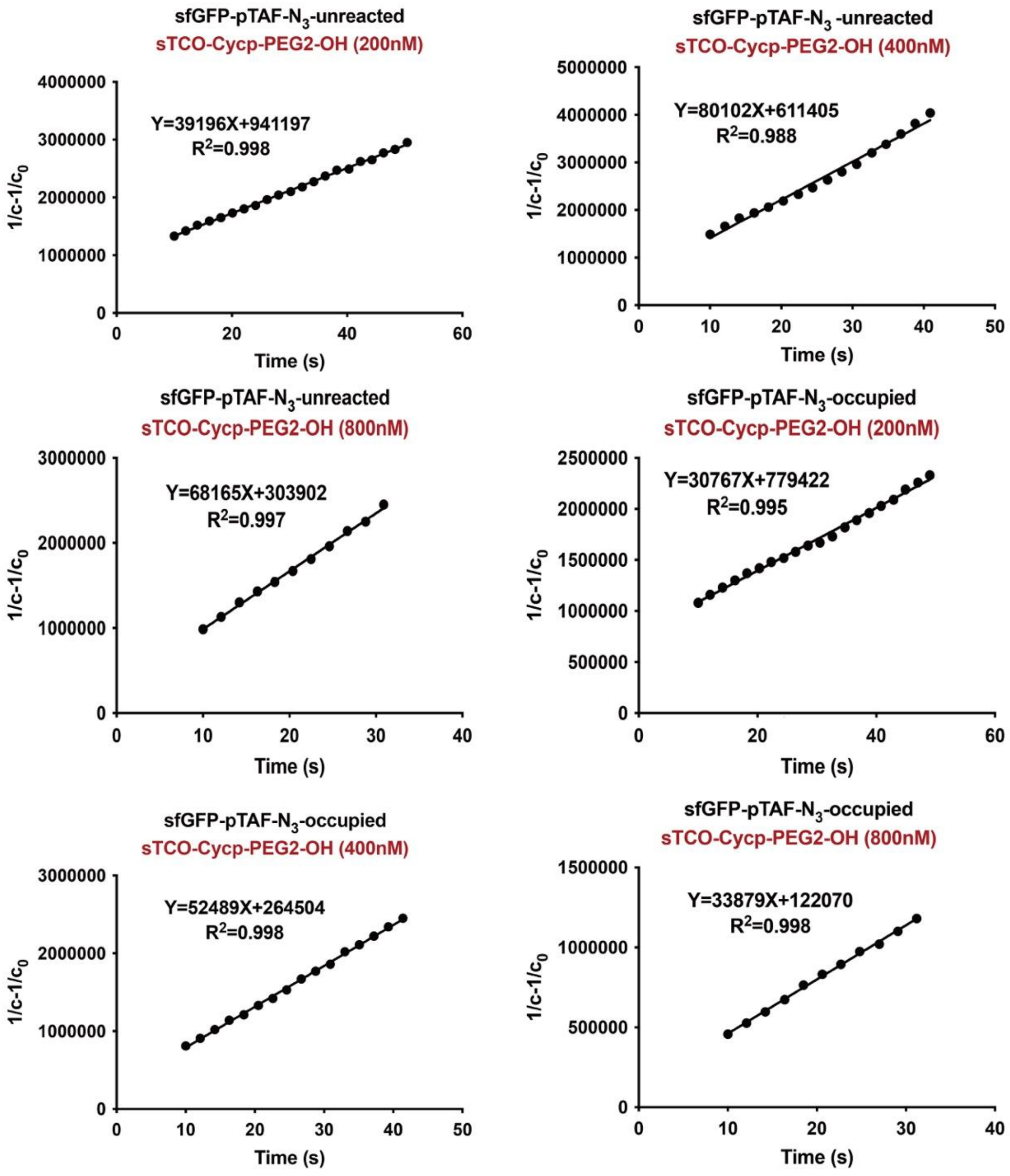

Supplementary Fig. 9. Plotting kinetics curve of $1/c-1/c_0$ with time for sfGFP-pTAF-N_3 (N_3 unreacted) and sfGFP-pTAF-N_3-DBCO-amine (N_3 occupied). After measuring to obtain standard curve of sfGFP-pTAF and sfGFP-pTAF-DBCO-amine though fluorescence change, the calibration curves were constructed to convert fluorescence intensity signal at each spot into $1/c-1/c_0$ (c: concentration of sfGFP-pTAF or sfGFP-pTAF-DBCO-amine at a certain time; c_0: the initial concentration of sfGFP-pTAF or sfGFP-pTAF-DBCO-amine), according to the integral formula $1/c-1/c_0 = kt$ of the rate equation of the second-order reaction when the reactant concentrations are the same, the slope obtained by plotting $1/c-1/c_0$ with t

(time) is the corresponding rate constant. It should be noted that the system is not a standard second-order reaction at a later time due to human error, so the starting points would be selected as much as possible. Source data are provided as a Source Data file.

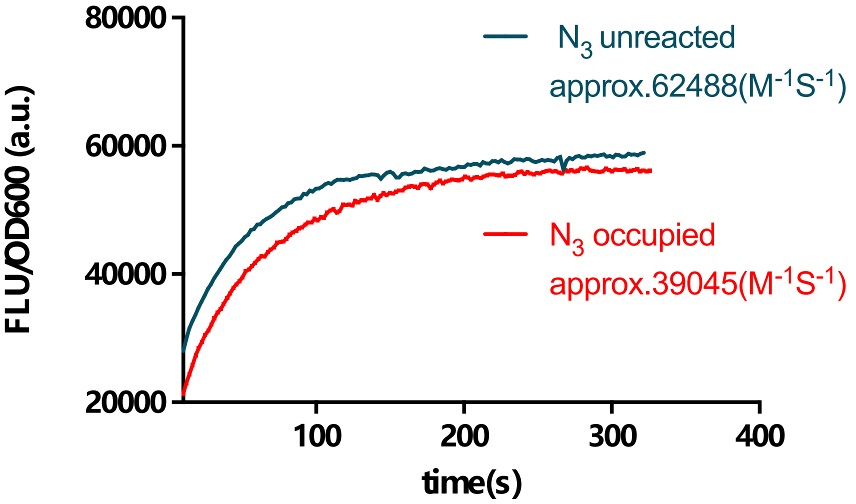

Supplementary Fig. 10. **Rate constants for reactions of sfGFP-Y151-pTAF and sfGFP-Y151-pTAF-DBCO-amine with sTCO-CycP-PEG2-OH.** The rate constants obtained in different systems at each concentration are averaged to obtain the final second-order reaction rate constant: azide unreacted (Approx. 62488 $M^{-1}S^{-1}$) and azide occupied (Approx. 39045 $M^{-1}S^{-1}$). Fluorescence is shown after normalization to the OD at 600 nm in arbitrary units (a.u.). Source data are provided as a Source Data file.

Supplementary Discussion

1. Measured second-order rate constants of azide in the presence of unreacted tetrazine or tetrazine occupied after reacting with TCO-small molecule or TCO-PEG at free amino acid level

To begin with, compounds pTAF-Biotin (Supplementary Fig. 11) and pTAF-PEG20K (Supplementary Fig. 11) were prepared by mixing pTAF amino acid (Supplementary Fig. 11) with TCO-PEG4-Biotin or bulky TCO-PEG20K with 1:1 stoichiometry, respectively, followed by purification using semi-preparative HPLC and

lyophilization. The reaction rate constants of free pTAF (20 μM) with DBCO-COOH (200 μM), pTAF-Biotin (200 μM) with DBCO-COOH (200 μM) and pTAF-PEG20K (2 mM) with DBCO-COOH (2 mM) respectively, were determined using HPLC analysis at 25 °C. Product peaks were detected by 2998 photodiode array detector at 254 nm (Waters), the peak area of reactants and products were taken at different time points over a period of 12-24 h using a 10-50% B gradient 10 min and 50-10% B gradient next 10 min. Peak areas were integrated for relative quantification using the Empower software (Waters).The second order rate constants of free pTAF, pTAF-Biotin, and pTAF-PEG20K with DBCO-COOH were then determined to be **0.70 ± 0.4 $M^{-1}s^{-1}$, 0.66 ± 0.03 $M^{-1}s^{-1}$ and 0.30 ± 0.02 $M^{-1}s^{-1}$**, respectively

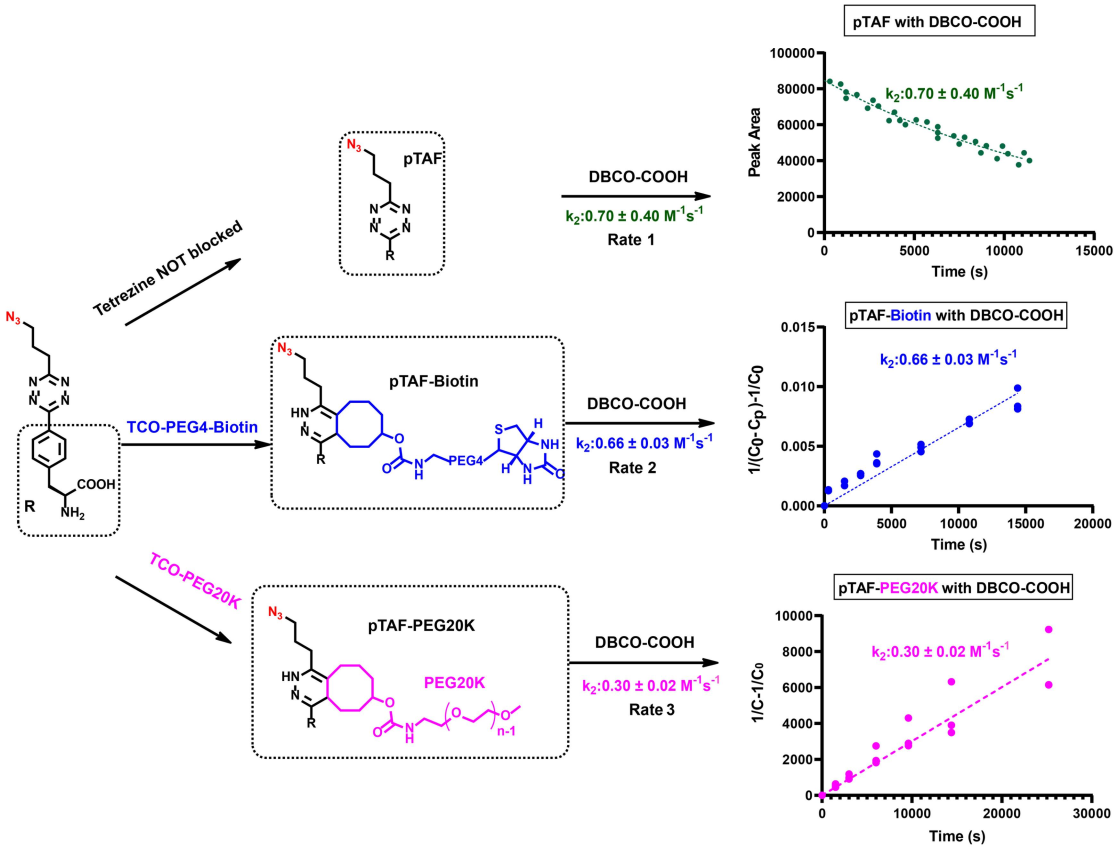

Supplementary Fig. 11. Measured second-order rate constants of free pTAF, pTAF-Biotin, and pTAF-PEG20K with DBCO-COOH respectively in MeOH at 25 °C. Note that the different ordinates were chose for integrating based on the different UV absorption strength about the reactants or products. The total peak

area of reactants or products were added and plotted against time and constructed the calibration curves to convert peak area at each spot into $1/c-1/c_0 = kt$ (c: concentration of reactants at a certain time; c_0: the initial concentration of reactants), $1/(c_0-c_p)-1/c_0= kt$ (c_0: the initial concentration of reactants; c_p: concentration of product at a certain time), OR fit to one phase exponential decay using the Prism software package. Note: No error bars appear for some points because of they are shorter than the size of the symbol. Source data are provided as a Source Data file. Data are presented as mean values ± s.d. (n = 3 biologically independent experiments).

2. Measured second-order rate constants of azide in the presence of unreacted tetrazine or tetrazine occupied after reacting with TCO-small molecule or TCO-PEG at proteins containing pTAF residue

IFN-α2b containing pTAF at the site of E42 was chosen as a model protein. To occupy the tetrazine position, TCO-PEG3-FITC (2 equivalents) and TCO-PEG20K (2 equivalents) were reacted with the tetrazine group of IFN-α2b-E42pTAF (1 equivalents), respectively and the resulting protein conjugates were purified to obtain IFN-α2b-PEG3-FITC and IFN-α2b-PEG20K. Next, to 40 μl 5 μM IFN-α2b-pTAF, 20 μM IFN-α2b-PEG3-FITC and 10 μM IFN-α2b-PEG20K in PBS respectively, 40 μl of 10, 40, 20 μM DBCO-CY5 in PBS was added and mixed at 25 °C. At each time point, 5 μl mixture was taken out and mixed with 5 μl 1M N-ethyl-N-hydroxy-ethanamine to quench the excessive DBCO-CY5. All the samples were then mixed with 4 μl of NuPAGE LDS sample buffer, heated at 95°C for 10 min and loaded onto 15 % Bis-Tris gels. The fluorescence change for conjugated products taken at different time points was measured and the fluorescence intensity was integrated for relative quantification by using Typhoon Imager. The rate constant of azide on IFN-α2b-pTAF, IFN-α2b-PEG3-FITC and IFN-α2b-PEG20K with DBCO-CY5 were determined to be 181.66 ± 31.32 $M^{-1}s^{-1}$ (Supplementary Fig. 12a) 746.27 ± 125.78 $M^{-1}s^{-1}$ (Supplementary Fig. 12b) and 177.60 ± 11.18 $M^{-1}s^{-1}$ (Supplementary Fig. 12c), respectively. Interestingly, the reaction rate of azide on IFN-α2b-PEG3-FITC, where

the tetrazine was occupied, was four-fold higher than the one on IFN-α2b-pTAF, where the tetrazine was unoccupied. It is possible that the π-π interaction or hydrophobic interaction between fluorescein and CY5 could accelerate the reaction. Most importantly, the reaction rate of azide on IFN-α2b-PEG20K (177.60 ± 11.18 $M^{-1}s^{-1}$) was identical to the reaction rate of azide on IFN-α2b-pTAF (181.66 ± 31.32 $M^{-1}s^{-1}$), indicating that the bulky PEG20K attaching the tetrazine group does not have a steric hindrance effect on the following azide-DBCO reaction.

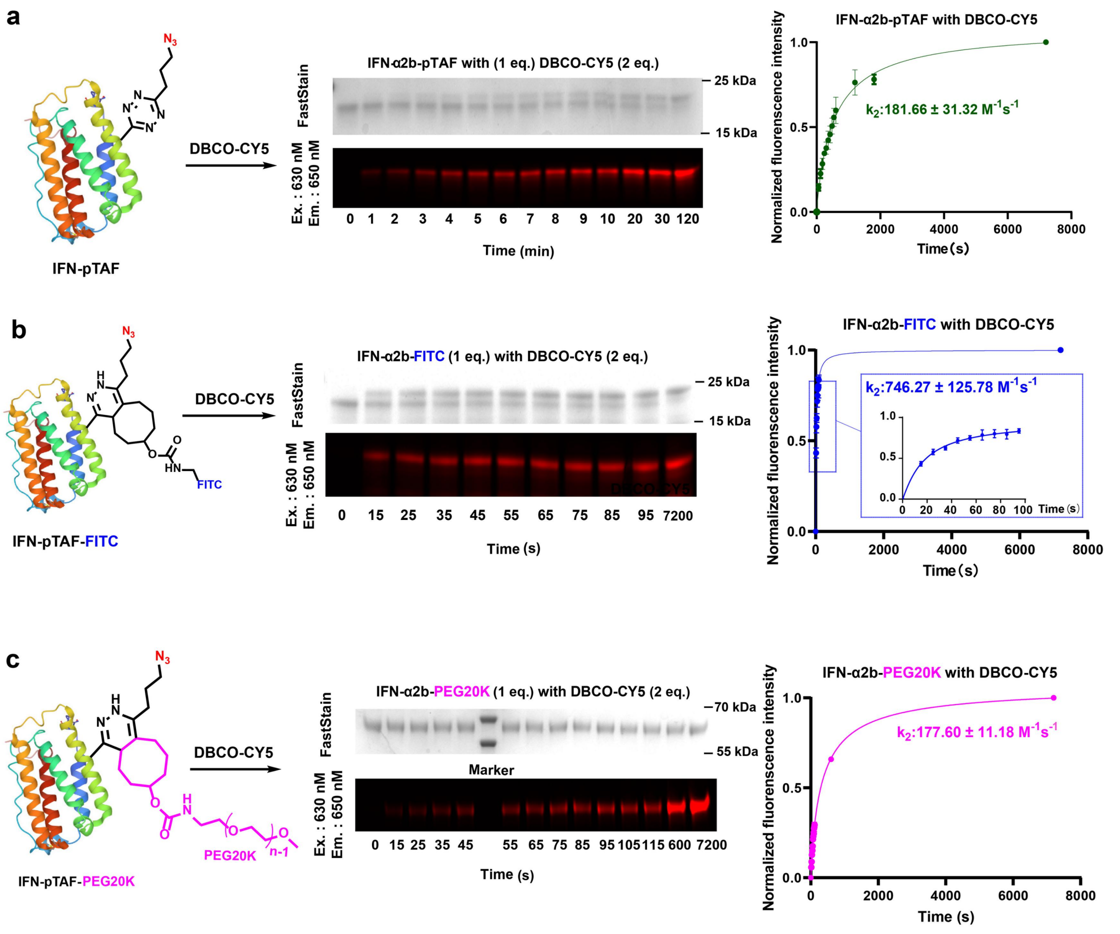

Supplementary Fig. 12. Second-order rate constants determination between azide and DBCO-CY5 on IFN-α2b-pTAF (a), IFN-α2b-PEG3-FITC (b) and IFN-α2b-PEG20K (c). The reaction rates were determined by time course fluorescent gel imaging using Typhoon. Data are presented as mean values ± s.d. (n = 3 biologically independent experiments). Note: No error bars appear for some points because of they are shorter than the size of the symbol. (IFN-α2b PDB ID: 2KZ1 [https://www.rcsb.org/structure/2KZ1]). Source data are provided as a Source Data file.

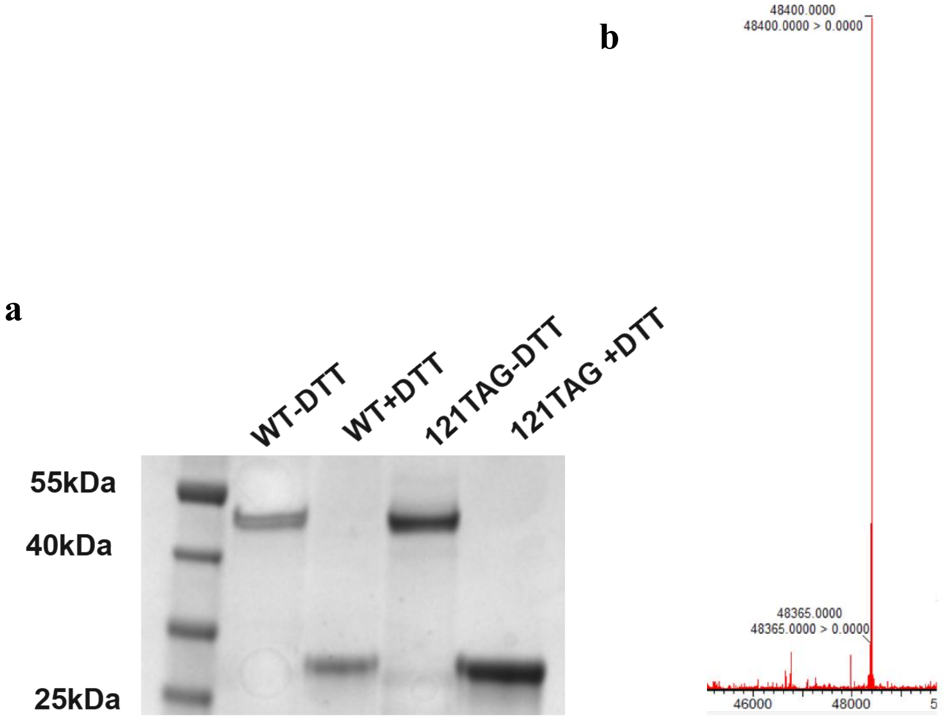

Supplementary Fig. 13. SDS-PAGE and High-resolution electrospray-ionization mass spectrum of J591 Fab. SDS-PAGE analysis (a) revealed that the wild type J591 Fab (High-resolution MS is 48400.000 kDa, b) and J591 Fab A121-pTAF migrates as a single band around 50 kDa in the absence of DTT, matching the calculated molecular weight of the J591 Fab. In addition, in the presence of DTT, the heavy and light chains of the J591 Fab migrate at 25 kDa, respectively. The purification experiments were repeated three times with reproducible results.

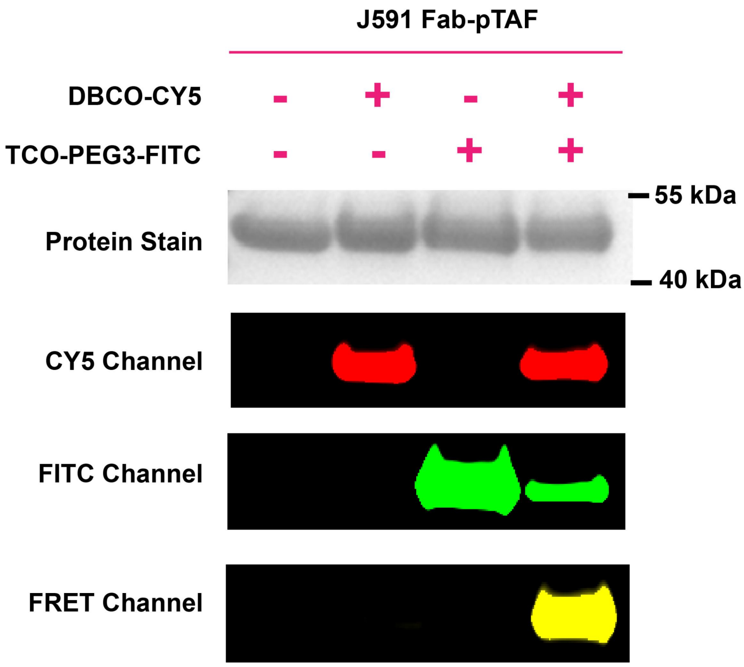

Supplementary Fig. 14. Fluorescence SDS-PAGE images of conjugate J591 Fab-pTAF upon treatment with DBCO-CY5 and/or TCO-PEG3-FITC. Purified Fab-pTAF (lane 1) was labelled with 5 equiv. DBCO-CY5, which yielded J591 Fab-DBCO-CY5 (lane 2), or to conjugate 2 equiv. TCO-PEG3-FITC to yield J591 Fab-TCO-PEG3-FITC (lane 3). J591 Fab-DBCO-CY5-TCO-PEG3-FITC conjugates was labelled with 5 equiv. DBCO-CY5 and TCO-PEG3-FITC via a one-pot reaction (lane 4). Denatured and reduced 12% SDS-PAGE gel. protein FastStain. Fluorescence conjugations were visualized by fluorescence imaging using a Typhoon Imager. Assays were repeated three times with reproducible results.

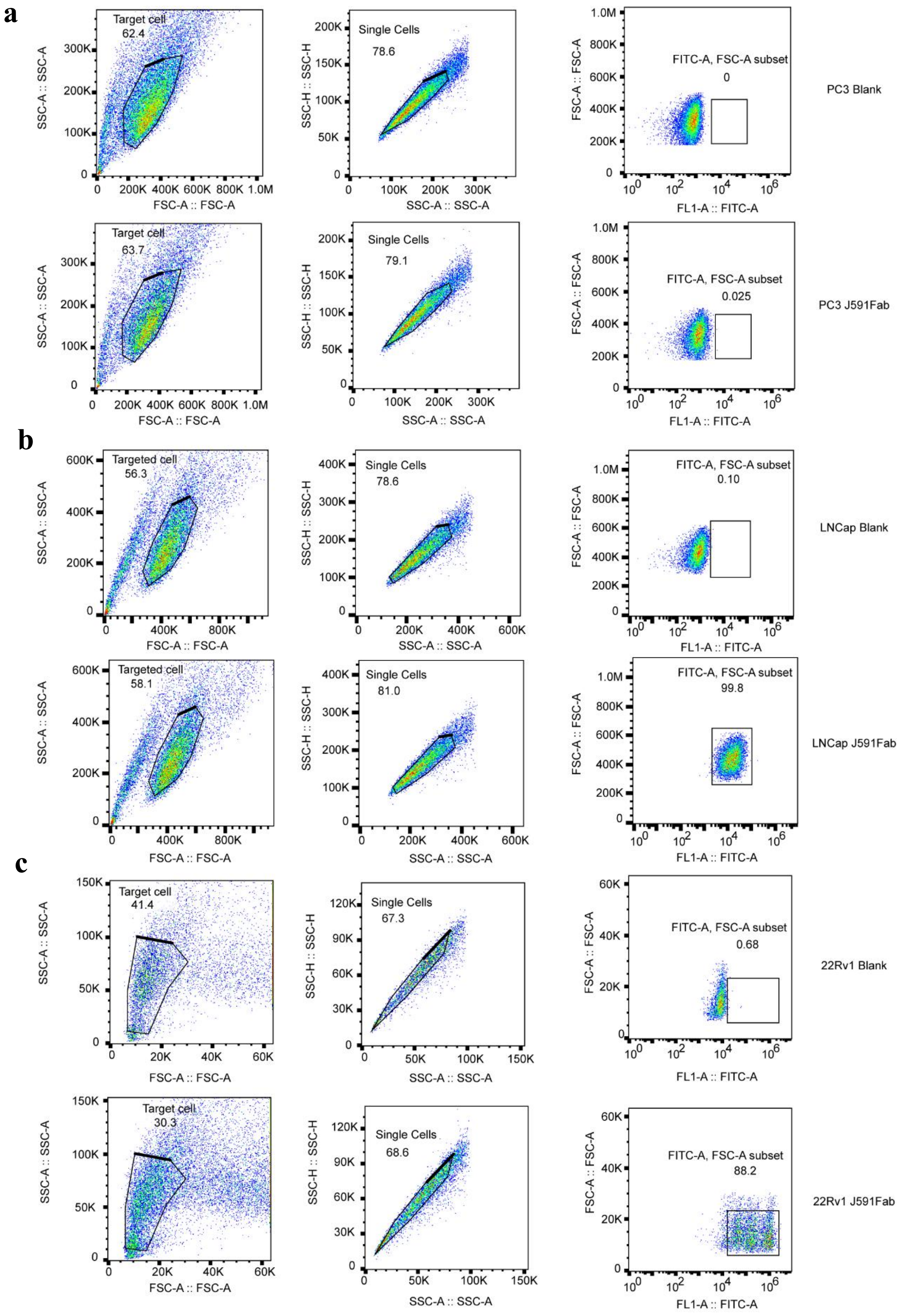

Supplementary Fig. 15. Flow cytometric gating strategy for the analysis of wild-type J591Fab binding to PC3 cells (a), LNCaP cells (b), and 22Rv1 cells (c). The cells were gated to exclude debris and doublets. LNCaP cells (PSMA+), and 22Rv1 cells (PSMA+) are gated in the FITC channel.

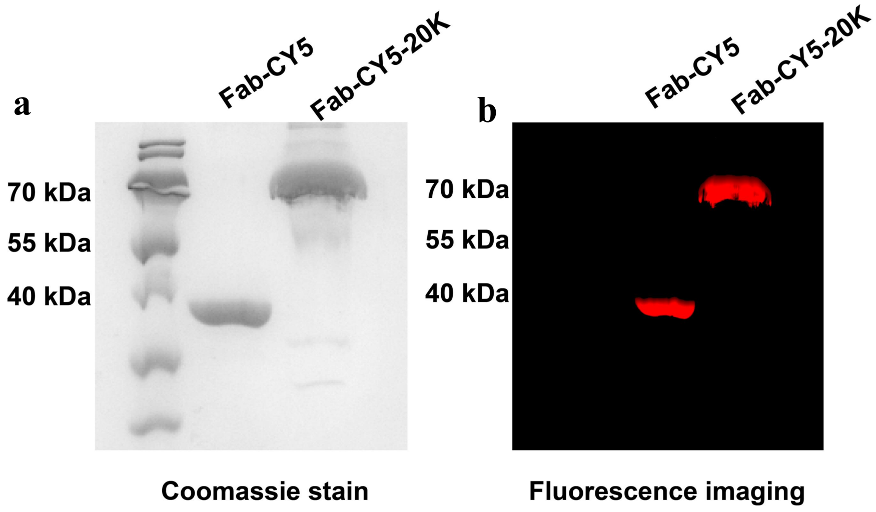

Supplementary Fig. 16. SDS–PAGE (a) and fluorescence imaging (b) analysis of J591 Fab-CY5 and J591 Fab-CY5-20K. The CY5 labeling efficiency was more than 90% by the ratio of the Cy5 concentration to the protein concentration, which was calculated by using the preset-program "Protein and Labels" of Nanodrop 2000c (Thermo Scientific) to measure the concentration of protein and CY5. Assays were repeated three times with reproducible results.

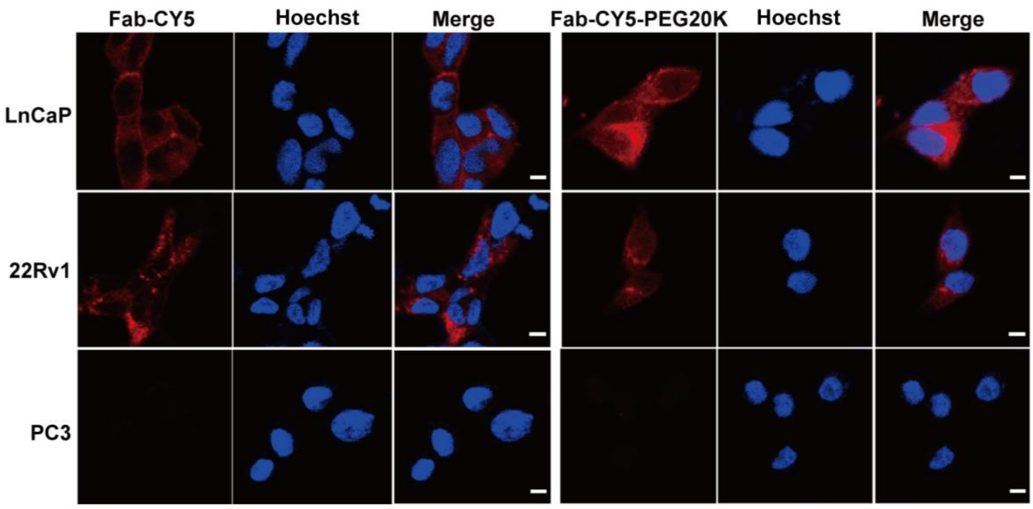

Supplementary Fig. 17. Binding of Fab-CY5 and Fab-CY5-PEG20K to LNCaP /22Rv1/PC3 cells was analyzed by Confocal Microscopy. Confocal microscopy images of PSMA-positive LNCaP /22Rv1 cells and PSMA-negative PC3 cells stained with CY5-conjugated J591Fab (red) and CY5-20K-conjugated J591Fab (red). The

nuclei were stained with Hoechst (blue). scale bar: 20 μm. Assays were repeated three times with reproducible results.

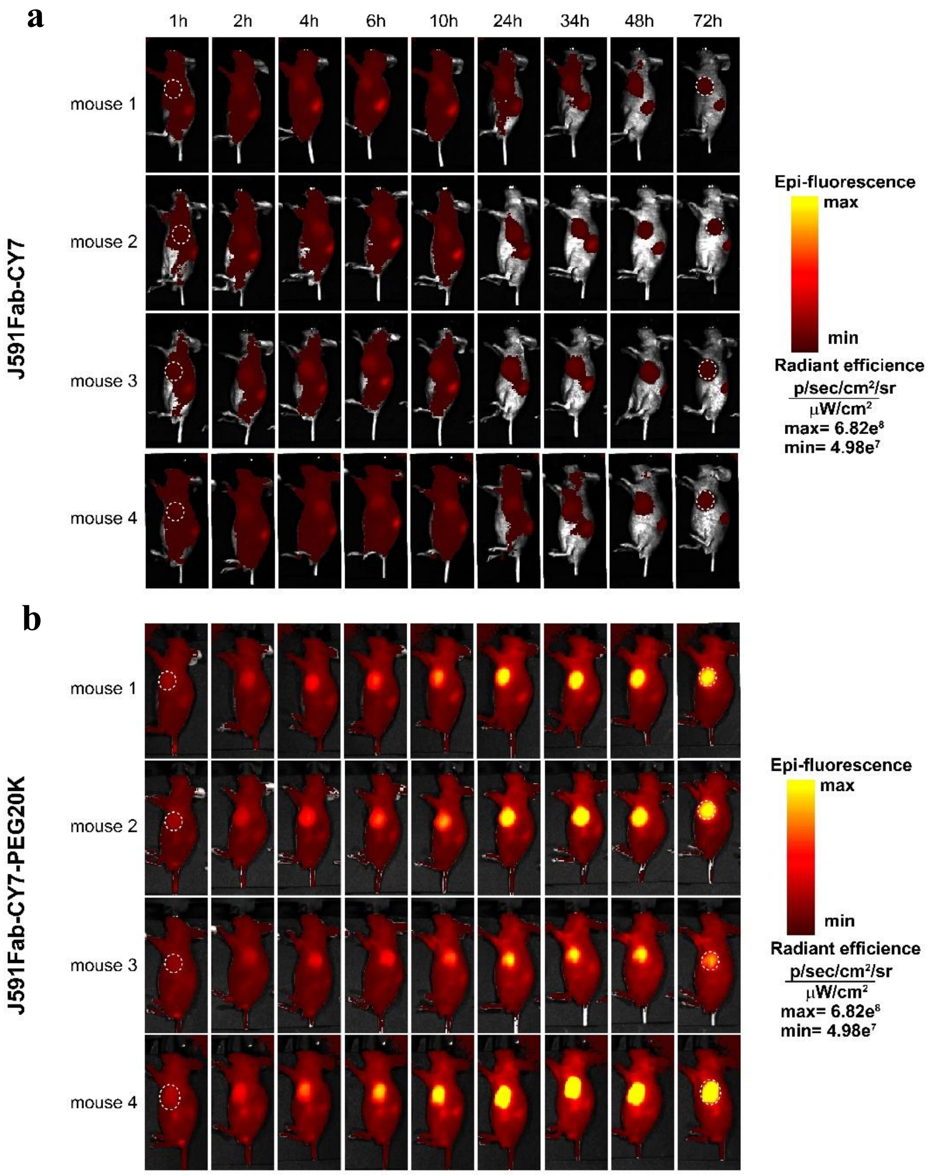

Supplementary Fig. 18. Fluorescence imaging of 22Rv1 tumors in vivo with Fab-CY7 (a) and Fab-CY7-PEG20K (b). Mice were intravenously injected with 60 μg Fab-CY7 or Fab-CY7-PEG20K, respectively. Images were acquired at 1h, 2h, 4h, 6h, 10h, 24h, 34h, 48h and 72h post injection. Every group has four independent mice.

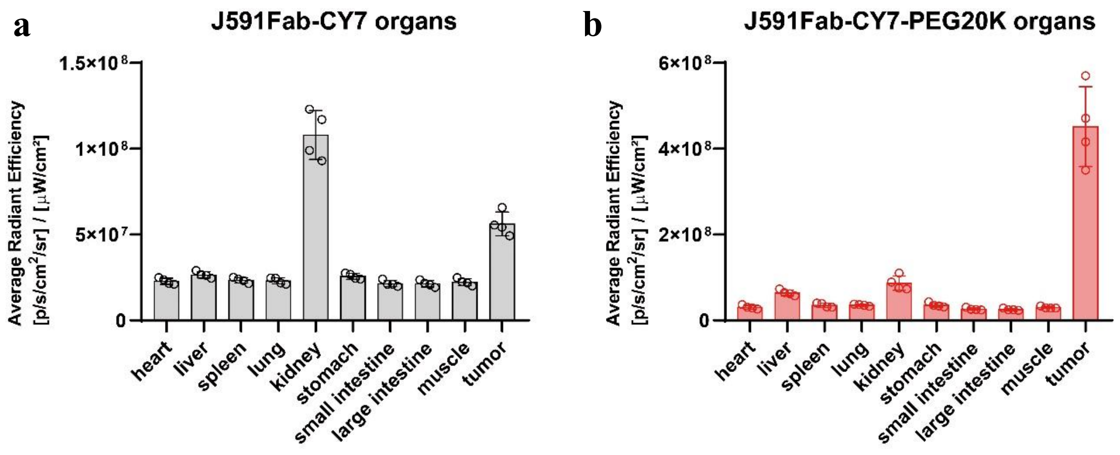

Supplementary Fig. 19. Ex vivo evaluation the fluorescence intensity of dissected organs. Ex vivo evaluation of dissected organs at 72 h of the J591Fab-CY7 (a) and J591Fab-CY7-PEG20K (b) groups. Data are shown presented as mean ± s.d (n=4 independent experiments). Source data are provided as a Source Data file.

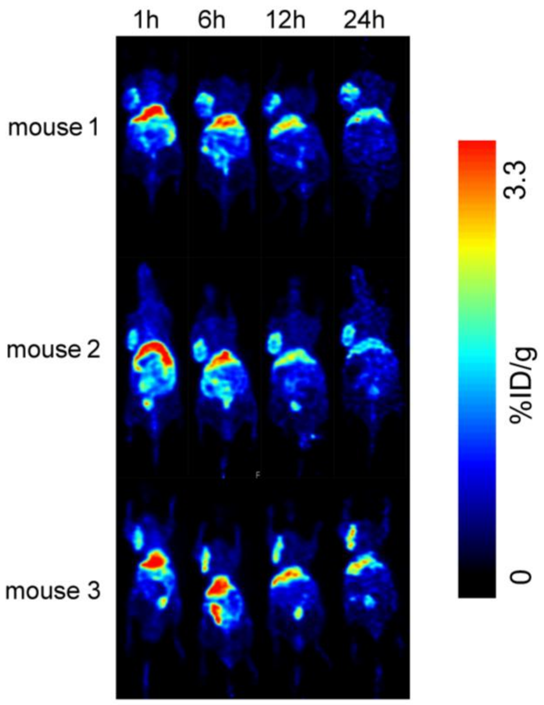

Supplementary Fig. 20. Micro-PET imaging of 22Rv1 tumors with ^{64}Cu labeled Fab-Cy5-NOTA. Images were acquired at 1 h, 6 h, 12 h and 24 h post injection. *N* = 3 mice for 22Rv1 group.

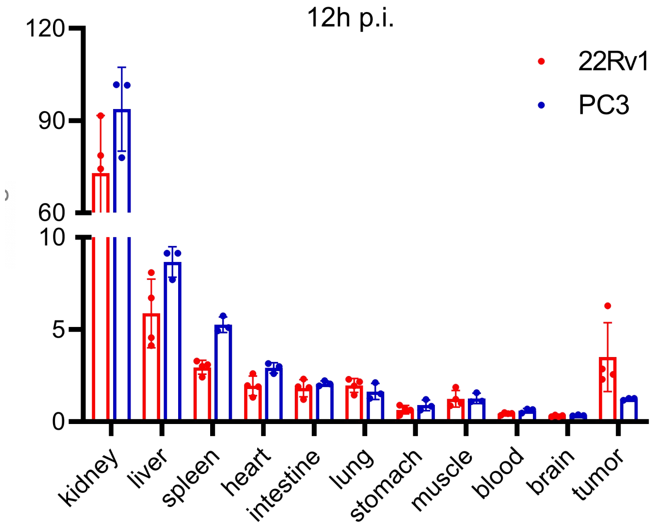

Supplementary Fig. 21. Bio-distribution of ^{64}Cu labeled Fab-CY5-NOTA at 12 h post injection. n = 3 mice for PC3 group and n = 4 mice for 22Rv1 group. Data are presented as mean value ± s.d. Source data are provided as a Source Data file.

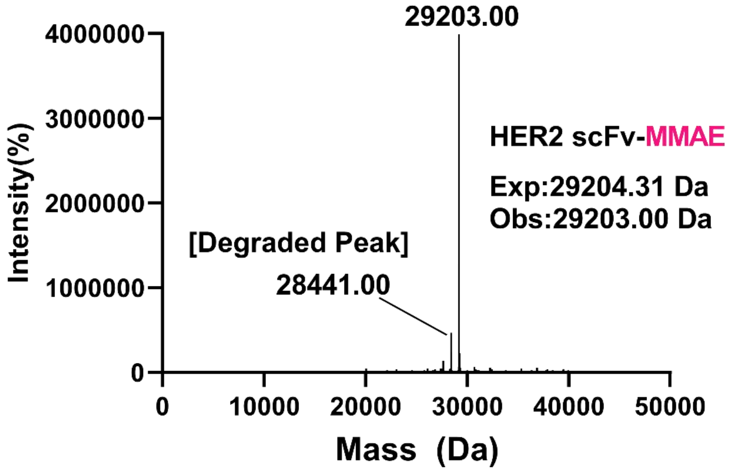

Supplementary Fig. 22. High-resolution electrospray-ionization mass spectrum of purified HER2-scFv-MMAE. Source data are provided as a Source Data file. Source data are provided as a Source Data file.

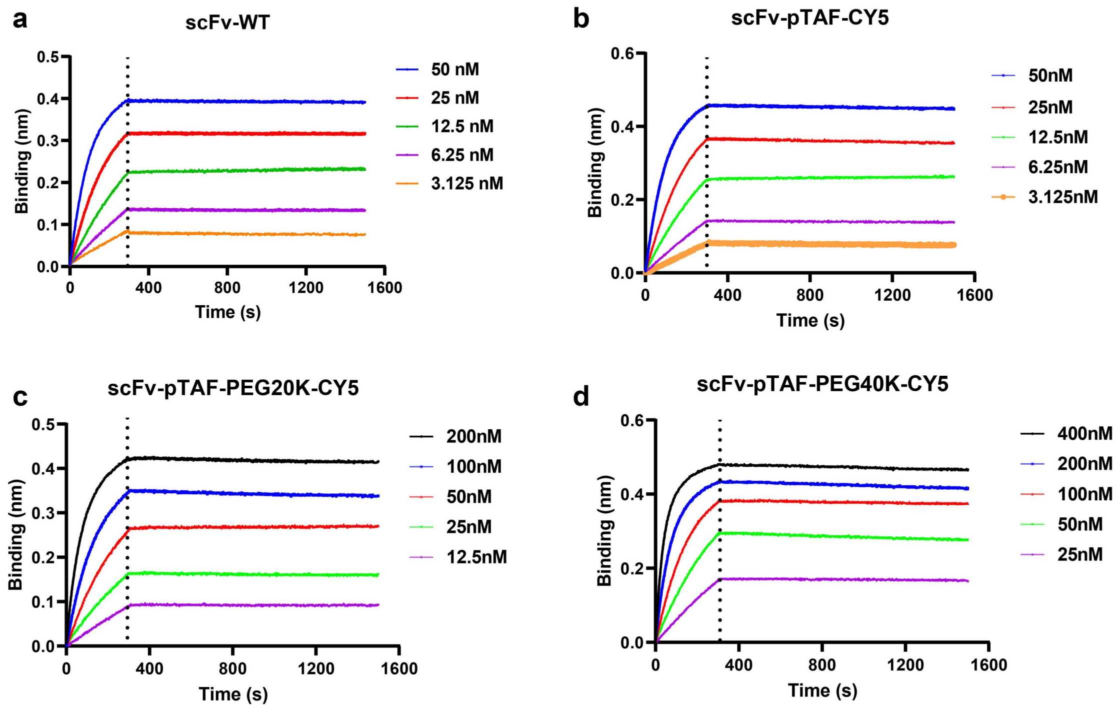

Supplementary Fig. 23. Biolayer interferometry analysis of HER2/ERBB2 protein with scFv-WT (a), scFv-pTAF-CY5 (b), scFv-pTAF-PEG20K-CY5 (c), and scFv-pTAF-PEG40K-CY5 (d). The experimental detail can be found in the method section. BLI experiments were performed on an Octet RED96e instrument (ForteBio Inc., Sartorius, Germany) using amine-reactive AR2G biosensors. Source data are provided as a Source Data file.

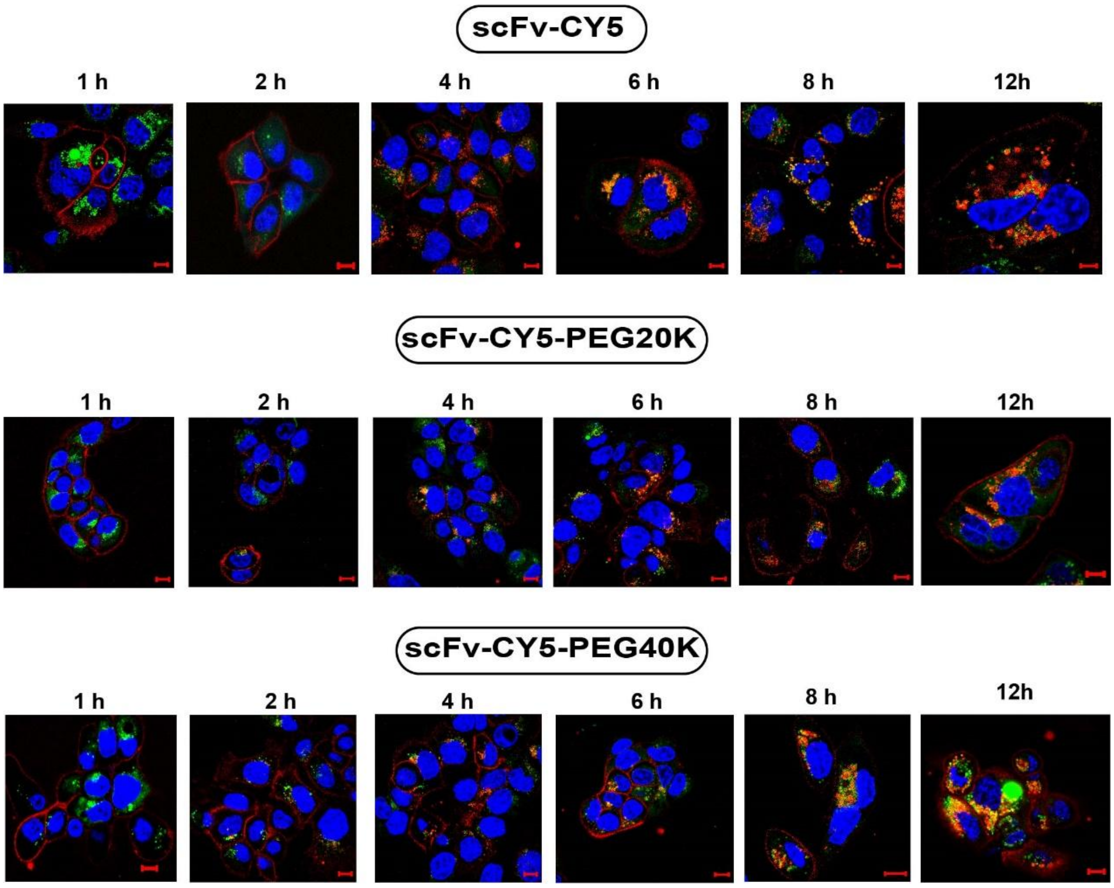

Supplementary Fig. 24. The confocal merge imaging showing the internalization of the scFv conjugates at different time points. Co-localization of scFv-CY5, scFv-CY5-PEG20K, and scFv-CY5-PEG40K with lysosome were imaged by merging pictures of CY5 staining and Lyso-Tracker Green staining**.** HCC1954 cells were seeded on glass bottom dishes (29 mm dish with 20 mm bottom well, 9×10^4 cells per dish) and were incubated at 37 °C under 5% CO_2 for 24 h. Then the cells were incubated with different scFv-CY5 conjugates at 20 μg/ml at 37°C for gradient time. Culture medium containing Hoechst 33342 (10 μg/ml) and Lyso-Tracker Green (75 nM) was added to the cells to stain cell nuclei and lysosome respectively for 30 min. Cells were washed with PBS to remove free dye and fresh culture medium was added. Glass bottom dishes were examined using Zeiss LSM880 Confocal microscope. Scale bars = 10 μm. Assays were repeated three times with reproducible results. Assays were repeated three times with reproducible results.

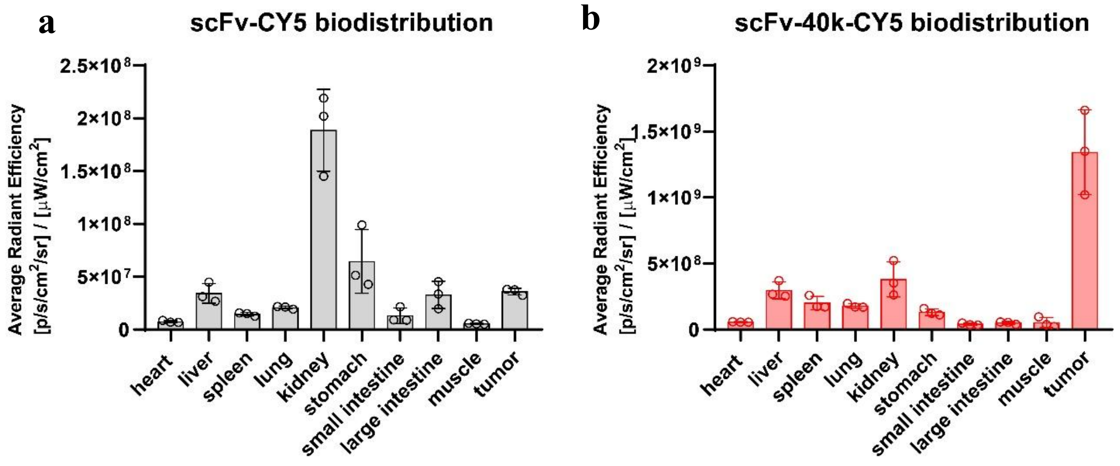

Supplementary Fig. 25. Ex vivo evaluation the fluorescence intensity of dissected organs. Ex vivo evaluation of dissected organs at 72 h p.i. of the scFv-CY5 (a) and scFv-CY5-PEG40K (b) groups. Data are shown presented as mean ± s.d (n=3 independent experiments). Source data are provided as a Source Data file.

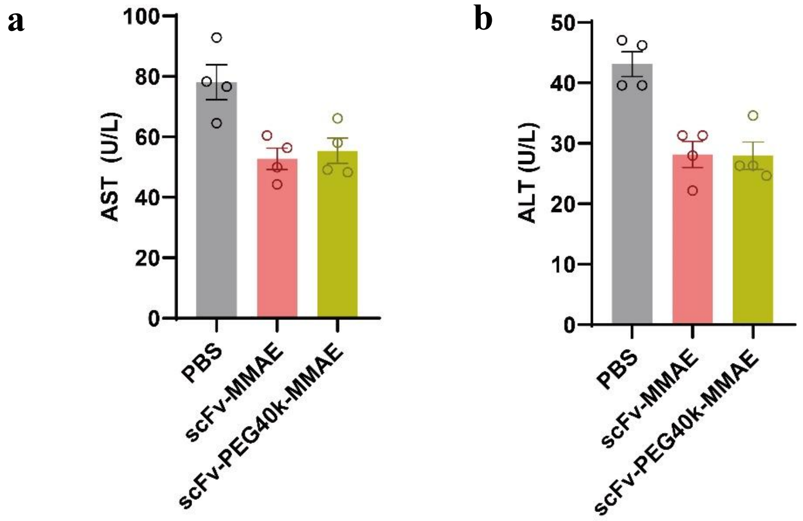

Supplementary Fig. 26. Changes in toxicological parameters upon conjugates dosing. Alanine aminotransferase (AST, a) and Aspartate aminotransferase (ALT, b) in the serum of BALB/c nude mice receiving 10 mg/kg treatment were determined by ELISA after PBS, scFv-MMAE, or scFv-MMAE-PEG40K treatment. Data are shown presented as mean ± s.d (n=4 independent experiments). Source data are provided as a Source Data file.

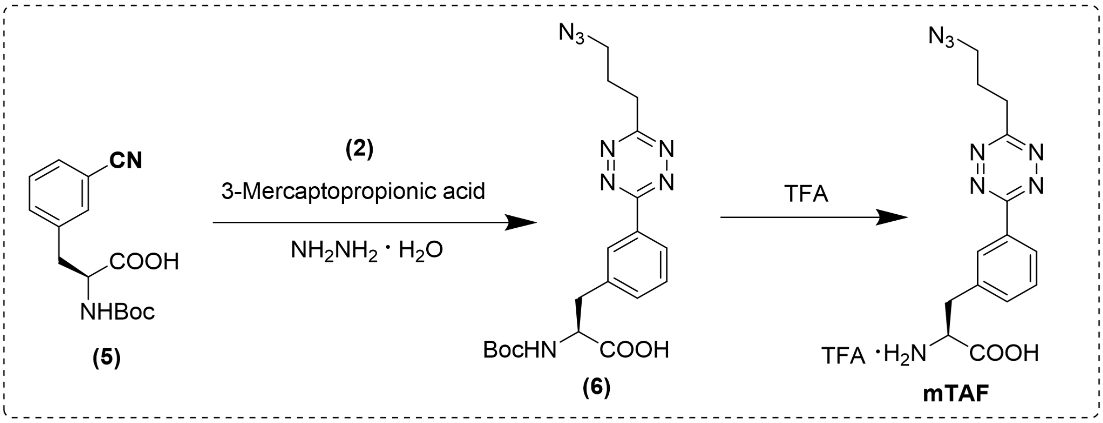

Supplementary Fig. 27. Synthetic route of mTAF. mTAF was synthesized by reaction of Boc-L-3-cyanophenylalanine (**5**) with 4-azidobutanenitrile (**2**) in the presence of hydrazine hydrate and 3-mercaptopropionic acid to form a 1,2-dihydrotetrazine, which was then oxidized and deprotected to give mTAF.

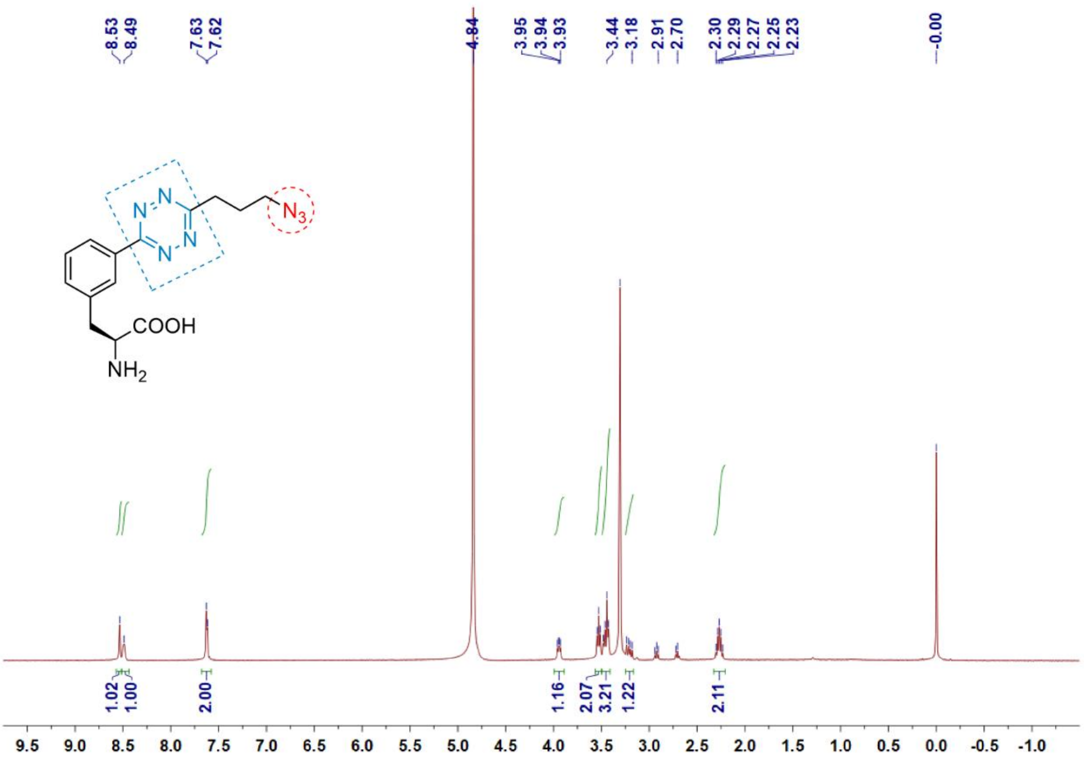

Supplementary Fig. 28. ^{1}H NMR spectra of mTAF

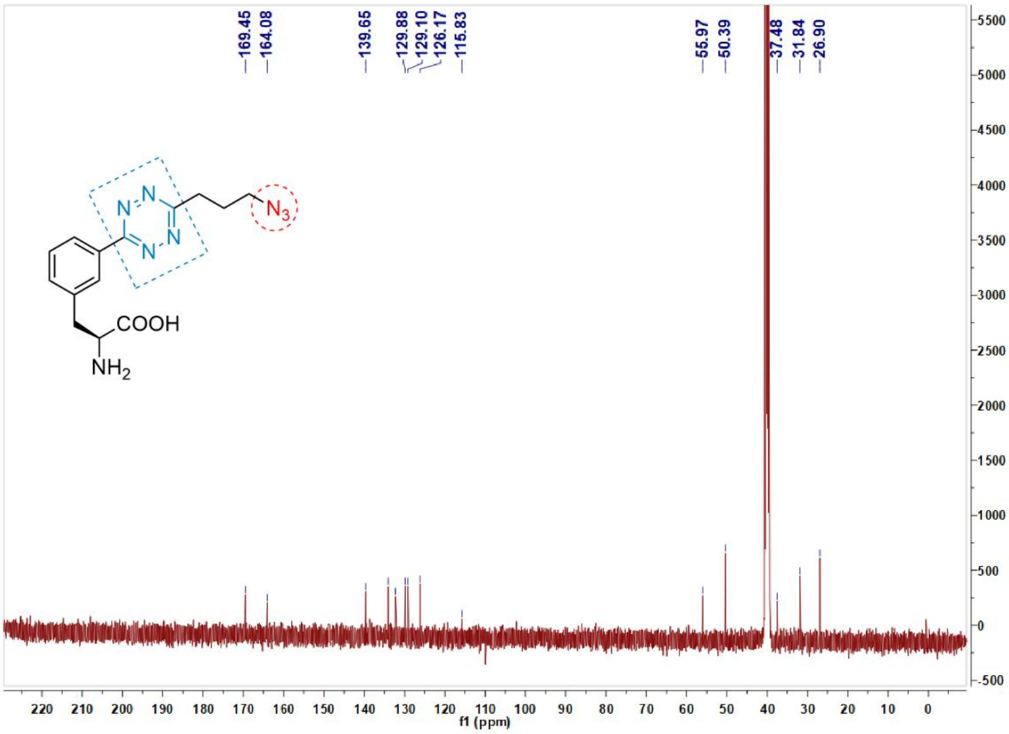

Supplementary Fig. 29. ^{13}C NMR spectra of mTAF

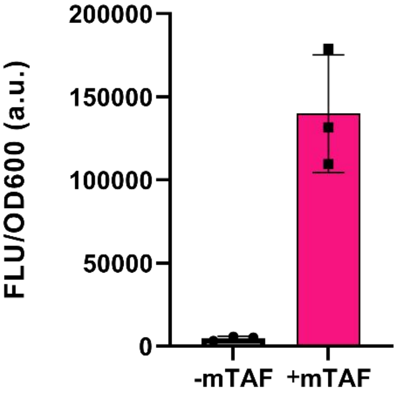

Supplementary Fig. 30. A quantitative sfGFP fluorescence assay in the absence or presence of 1 mM mTAF. SfGFP bearing amber codon was co-expressed with R2-74RS/tRNA pair containing plasmid. Samples were collected after 12 hours of cultivation. Fluorescence is shown after normalization to the OD at 600 nm in arbitrary units (a.u.). Data are presented as mean values ± s.d. (n = 3 biologically independent experiments).

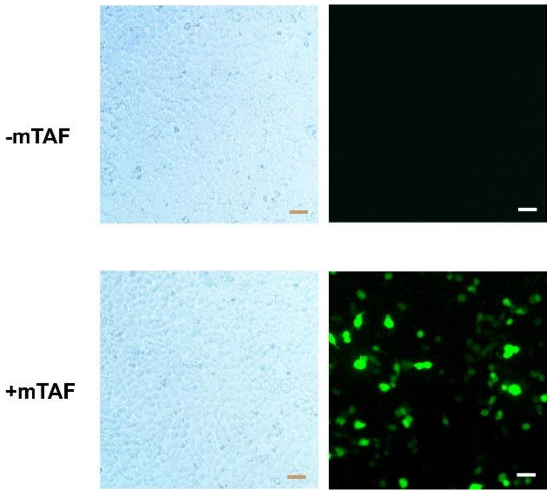

Supplementary Fig. 31. To site-specifically incorporate mTAF with R2-74-RS in HEK293T cell in the presence of 100 µM ncAAs. Assays were repeated three times with reproducible results. Scale bars = 50 µm.

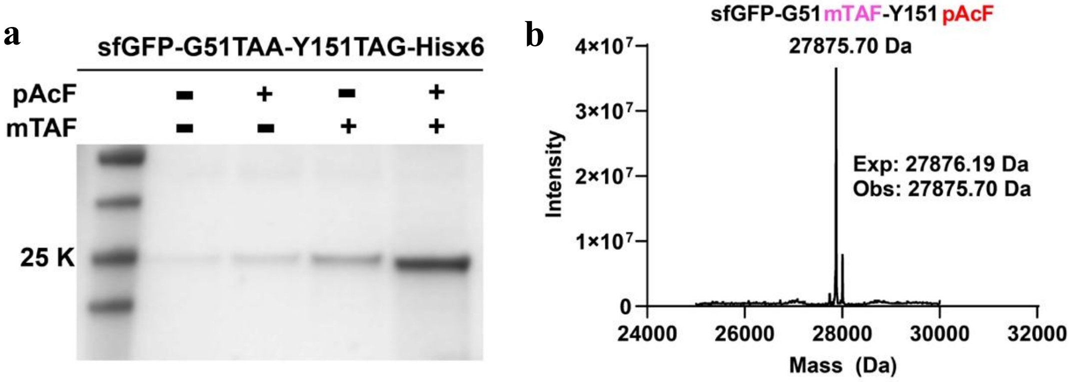

Supplementary Fig. 32. SDS–PAGE analysis of the orthogonality of the dual-encoding systems. Full-length sfGFP could be expressed in the presence of both the two types ncAAs-pAcF and mTAF (a); High-resolution electrospray-ionization mass spectrum of purified full-length sfGFP containing mTAF and pAcF ncAAs (b). The purification experiments were repeated three times with reproducible results.

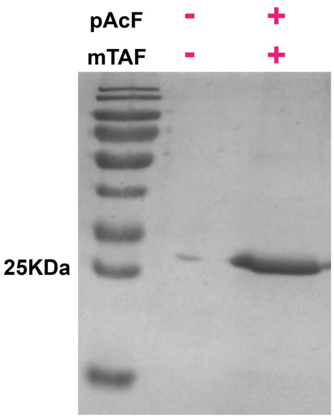

Supplementary Fig. 33. SDS–PAGE analysis of the HER2-scFv-S9pAcF-K42mTAF in the presence of pAcF and mTAF. Proteins were purified from *E. coli* cell lysates migrated as a single band at about 27 kDa. The purification experiments were repeated three times with reproducible results.

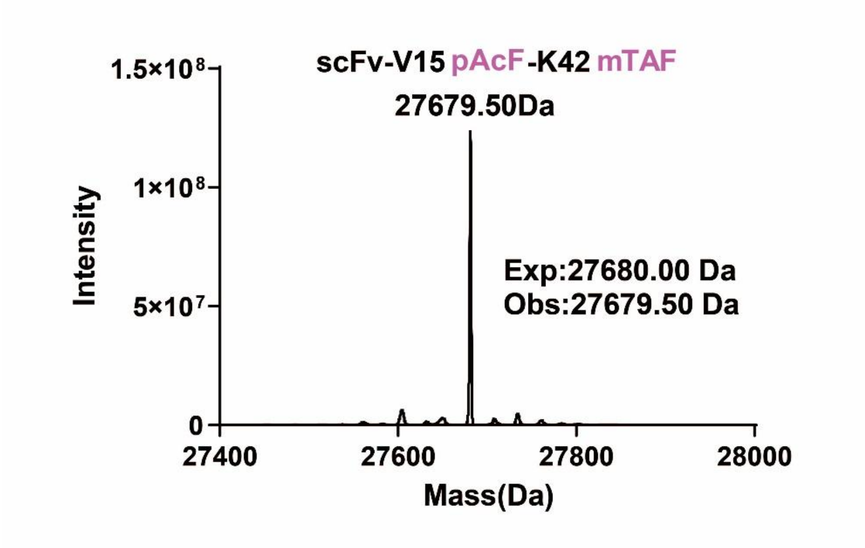

Supplementary Fig. 34. High-resolution electrospray-ionization mass spectrum of purified scFv-V15pAcF-K42mTAF.

Supplementary Note

Supplementary Gene and Protein Sequence:

sfGFP-WT (protein):

MSKGEELFTGVVPILVELDGDVNGHKFSVRGEGEGDATNGKLTLKFICTTGKL
PVPWPTLVTTLTYGVQCFSRYPDHMKRHDFFKSAMPEGYVQERTISFKDDGT
YKTRAEVKFEGDTLVNRIELKGIDFKEDGNILGHKLEYNFNSHNVYITADKQK
NGIKANFKIRHNVEDGSVQLADHYQQNTPIGDGPVLLPDNHYLSTQSVLSKD
PNEKRDHMVLLEFVTAAGITHGMDELYKGSHHHHHH

sfGFP-WT (DNA):

ATGAGCAAAGGAGAAGAACTTTTCACTGGAGTTGTCCCAATTCTTGTTGAA
TTAGATGGTGATGTTAATGGGCACAAATTTTCTGTCCGTGGAGAGGGTGAA
GGTGATGCTACAAACGGAAAACTCACCCTTAAATTTATTTGCACTACTGGA
AAACTACCTGTTCCGTGGCCAACACTTGTCACTACTCTGACCTATGGTGTTC
AATGCTTTTCCCGTTATCCGGATCACATGAAACGGCATGACTTTTTCAAGAG
TGCCATGCCCGAAGGTTATGTACAGGAACGCACTATATCTTTCAAAGATGAC
GGGACCTACAAGACGCGTGCTGAAGTCAAGTTTGAAGGTGATACCCTTGTT
AATCGTATCGAGTTAAAGGGTATTGATTTTAAAGAAGATGGAAACATTCTTG
GACACAAACTCGAGTACAACTTTAACTCACACAATGTATATATCACGGCAG
ACAAACAAAAGAATGGAATCAAAGCTAACTTCAAAATTCGCCACAACGTT
GAAGATGGTTCCGTTCAACTAGCAGACCATTATCAACAAAATACTCCAATT
GGCGATGGCCCTGTCCTTTTACCAGACAACCATTACCTGTCGACACAATCT
GTCCTTTCGAAAGATCCCAACGAAAAGCGTGACCACATGGTCCTTCTTGAG
TTTGTAACTGCTGCTGGGATTACACATGGCATGGATGAGCTCTACAAAGGAT
CCCACCACCACCACCACCAC

J591 Fab light chain-WT (protein):

MKKNIAFLLASMFVFSIATNAYADIVMTQSPSSLSASVGDRVTITCKASQDVGT
AVDWYQQKPGKAPKLLIYWASTRHTGVPDRFTGSGSGTDFTLTISSLQPEDFA

DYFCQQYNSYPLTFGGGTKLEIKRTVAAPSVFIFPPSDEQLKSGTASVVCLLNN
FYPREAKVQWKVDNALQSGNSQESVTEQDSKDSTYSLSSTLTLSKADYEKHK
VYACEVTHQGLSSPVTKSFNRGEC

J591 Fab light chain-WT (DNA):

ATGAAAAAGAATATCGCATTTCTTCTTGCTAGCATGTTCGTTTTTTCTATTGC
TACAAACGCATACGCTGACATCGTGATGACCCAGTCCCCCTCCTCCCTGTCT
GCCTCCGTGGGCGACAGAGTGACCATCACATGCAAGGCCTCCCAGGATGT
GGGCACCGCCGTGGACTGGTATCAGCAGAAGCCTGGCAAGGCCCCTAAGC
TGCTGATCTACTGGGCCTCCACCAGACACACCGGCGTGCCTGACAGATTCA
CCGGCTCCGGCTCTGGCACCGACTTCACCCTGACCATCTCCAGCCTGCAGC
CTGAGGACTTCGCCGACTACTTCTGCCAGCAGTACAACTCCTACCCTCTGA
CCTTCGGCGGAGGCACCAAGCTGGAAATCAAACGAACTGTGGCTGCACCA
TCTGTCTTCATCTTCCCGCCATCTGATGAGCAGTTGAAATCTGGAACTGCCT
CTGTCGTGTGCCTGCTGAATAACTTCTATCCCAGAGAGGCCAAAGTACAGT
GGAAGGTGGATAACGCCCTCCAATCGGGTAACTCCCAGGAGAGTGTCACA
GAGCAGGACAGCAAGGACAGCACCTACAGCCTCAGCAGCACCCTGACGCT
GAGCAAAGCAGACTACGAGAAACACAAAGTCTACGCCTGCGAAGTCACCC
ATCAGGGCCTGTCCTCGCCCGTCACAAAGAGCTTCAACAGGGGAGAGTGT

J591 Fab heavy chain-WT (protein):

MKKNIAFLLASMFVFSIATNAYAEVQLVQSGAEVKKPGASVKISCKTSGYTFT
EYTIHWVKQASGKGLEWIGNINPNNGGTTYNQKFEDRATLTVDKSTSTAYME
LSSLRSEDTAVYYCAAGWNFDYWGQGTTVTVSSASTKGPSVFPLAPSSKSTS
GGTAALGCLVKDYFPEPVTVSWNSGALTSGVHTFPAVLQSSGLYSLSSVVTVP
SSSLGTQTYICNVNHKPSNTKVDKKVEPKSCDKTHTAAALEHHHHHH

J591 Fab heavy chain-WT (DNA):

ATGAAAAAGAATATCGCATTTCTTCTTGCATCTATGTTCGTTTTTTCTATTGC
TACAAACGCGTACGCTGAAGTGCAGCTGGTGCAGTCTGGCGCCGAAGTGA

AGAAACCTGGCGCCTCCGTGAAGATCTCCTGCAAGACCTCCGGCTACACCT
TCACCGAGTACACCATCCACTGGGTGAAACAGGCCTCCGGCAAGGGCCTG
GAATGGATCGGCAACATCAACCCTAACAACGGCGGCACCACCTACAACCA
GAAGTTCGAGGACCGGGCCACCCTGACCGTGGACAAGTCCACCTCCACCG
CCTACATGGAACTGTCCTCCCTGCGGTCTGAGGACACCGCCGTGTACTACT
GCGCCGCTGGCTGGAACTTCGACTACTGGGGCCAGGGCACCACAGTGACA
GTCTCGAGCGCCTCCACCAAGGGCCCATCGGTCTTCCCCCTGGCACCCTCC
TCCAAGAGCACCTCTGGGGGCACAGCGGCCCTGGGCTGCCTGGTCAAGGA
CTACTTCCCCGAACCGGTGACGGTGTCGTGGAACTCAGGCGCCCTGACCA
GCGGCGTGCACACCTTCCCGGCTGTCCTACAGTCCTCAGGACTCTACTCCC
TCAGCAGCGTGGTGACTGTGCCCTCTAGCAGCTTGGGCACCCAGACCTACA
TCTGCAACGTGAATCACAAGCCCAGCAACACCAAGGTGGACAAGAAAGTT
GAGCCCAAATCTTGTGACAAAACTCACACAGCGGCCGCACTCGAGCACCA
CCACCACCACCAC

Anti-HER2-scFv-WT (protein):

MKKNIAFLLASMFVFSIATNAYADIQMTQSPSSLSASVGDRVTITCRASQDVNT
AVAWYQQKPGKAPKLLIYSASFLYSGVPSRFSGSRSGTDFTLTISSLQPEDFATY
YCQQHYTTPPTFGQGTKLEIKRT**<u>GSTSGSGKPGSGEGS</u>**EVQLVESGGGLVQP
GGSLRLSCAASGFNIKDTYIHWVRQAPGKGLEWVARIYPTNGYTRYADSVKG
RFTISADTSKNTAYLQMNSLRAEDTAVYYCSRWGGDGFYAMDYWGQGTLVT
VSSLEHHHHHH

Anti-HER2-scFv-WT (DNA):

ATGAAAAAGAATATCGCATTTCTTCTTGCTAGCATGTTCGTTTTTTCTATTGC
TACAAACGCATACGCTGACATCCAGATGACCCAGTCTCCATCCTCCCTGTCT
GCATCTGTAGGAGACAGAGTCACCATCACTTGCCGGGCAAGTCAGGATGT
GAATACCGCGGTCGCATGGTATCAGCAGAAACCAGGGAAAGCCCCTAAGC
TCCTGATCTATTCTGCATCCTTCTTGTATAGTGGGGTCCCATCAAGGTTCAGT
GGCAGTAGATCTGGGACAGATTTCACTCTCACCATCAGCAGTCTGCAACCT

GAAGATTTTGCAACTTACTACTGTCAACAGCATTACACTACCCCTCCGACGT
TCGGCCAAGGTACCAAGCTTGAGATCAAACGAACTGGCTCCACCAGCGGC
AGCGGCAAGCCAGGCTCCGGCGAAGGCAGCGAGGTGCAGCTGGTGGAGT
CTGGAGGAGGCTTGGTCCAGCCTGGGGGGTCCCTGAGACTCTCCTGTGCA
GCCTCTGGGTTCAATATTAAGGACACTTACATCCACTGGGTCCGCCAGGCT
CCAGGGAAGGGGCTGGAGTGGGTCGCACGTATTTATCCTACCAATGGTTAC
ACACGCTACGCAGACTCCGTGAAGGGCCGATTCACCATCTCCGCAGACACT
TCCAAGAACACGGCGTATCTTCAAATGAACAGCCTGAGAGCCGAGGACAC
GGCCGTGTATTACTGTTCGAGATGGGGCGGTGACGGCTTCTATGCCATGGA
CTACTGGGGCCAAGGAACCCTGGTCACCGTCTCCTCACTCGAGCACCACC
ACCACCACCAC

pTAFRS (protein):

MDEFEMIKRNTSEIISEEELREVLKKDEKSAGIGFEPSGKIHLGHYLQIKKMIDL
QNAGFDIIIALADLHAYLNQKGELDEIRKIGDYNKKVFEAMGLKAKYVYGSE
SDLDKDYTLNVYRLALKTTLKRARRSMELIAREDENPKVAEVIYPIMQVNGIH
YGGVDVAVGGMEQRKIHMLARELLPKKVVCIHNPVLTGLDGEGKMSSSKGN
FIAVDDSPEEIRAKIKKAYCPAGVVEGNPIMEIAKYFLEYPLTIKRPEKFGGDLT
VNSYEELESLFKNKELHPMDLKNAVAEELIKILEPIRKRL

pTAFRS (DNA):

ATGGACGAATTTGAAATGATAAAGAGAAACACATCTGAAATTATCAGCGAG
GAAGAGTTAAGAGAGGTTTTAAAAAAAGATGAAAAATCTGCTGGCATAGG
TTTTGAACCAAGTGGTAAAATACATTTAGGGCATTATCTCCAAATAAAAAAG
ATGATTGATTTACAAAATGCTGGATTTGATATAATTATAGCATTGGCTGATTT
ACACGCCTATTTAAACCAGAAAGGAGAGTTGGATGAGATTAGAAAAATAG
GAGATTATAACAAAAAAGTTTTTGAAGCAATGGGGTTAAAGGCAAAATATG
TTTATGGAAGTGAAAGTGATCTTGATAAGGATTATACACTGAATGTCTATAG
ATTGGCTTTAAAAACTACCTTAAAAAGAGCAAGAAGGAGTATGGAACTTAT
AGCAAGAGAGGATGAAAATCCAAAGGTTGCTGAAGTTATCTATCCAATAAT

GCAGGTTAATGGAATTCATTATGGAGGCGTTGATGTTGCAGTTGGAGGGAT
GGAGCAGAGAAAAATACACATGTTAGCAAGGGAGCTTTTACCAAAAAAGG
TTGTTTGTATTCACAACCCTGTCTTAACGGGTTTGGATGGAGAAGGAAAGA
TGAGTTCTTCAAAAGGGAATTTTATAGCTGTTGATGACTCTCCAGAAGAGA
TTAGGGCTAAGATAAAGAAAGCATACTGCCCAGCTGGAGTTGTTGAAGGA
AATCCAATAATGGAGATAGCTAAATACTTCCTTGAATATCCTTTAACCATAAA
AAGGCCAGAAAAATTTGGTGGAGATTTGACAGTTAATAGCTATGAGGAGTT
AGAGAGTTTATTTAAAAATAAGGAATTGCATCCAATGGATTTGAAGAATGCT
GTAGCTGAAGAACTTATAAAGATTTTAGAGCCAATTAGAAAGAGATTA

pAcFRS (protein):

MDEFEMIKRNTSEIISEEELREVLKKDEKSAVIGFEPSGKIHLGHYLQIKKMIDL
QNAGFDIIIYLADLHAYLNQKGELDEIRKIGDYNKKVFEAMGLKAKYVYGSE
HGLDKDYTLNVYRLALKTTLKRARRSMELIAREDENPKVAEVIYPIMQVNGI
HYQGVDVAVGGMEQRKIHMLARELLPKKVVCIHNPVLTGLDGEGKMSSSKG
NFIAVDDSPEEIRAKIKKAYCPAGVVEGNPIMEIAKYFLEYPLTIKRPEKFGGDL
TVNSYEELESLFKNKELHPMRLKNAVAEELIKILEPIRKRL

pAcFRS (DNA):

ATGGACGAATTTGAAATGATAAAGAGAAACACATCTGAAATTATCAGCGAG
GAAGAGTTAAGAGAGGTTTTAAAAAAAGATGAAAAATCTGCTGTTATAGGT
TTTGAACCAAGTGGTAAAATACATTTAGGGCATTATCTCCAAATAAAAAAGA
TGATTGATTTACAAAATGCTGGATTTGATATAATTATATATTTGGCTGATTTAC
ACGCCTATTTAAACCAGAAAGGAGAGTTGGATGAGATTAGAAAAATAGGA
GATTATAACAAAAAAGTTTTTGAAGCAATGGGGTTAAAGGCAAAATATGTT
TATGGAAGTGAACATGGTCTTGATAAGGATTATACACTGAATGTCTATAGAT
TGGCTTTAAAAACTACCTTAAAAAGAGCAAGAAGGAGTATGGAACTTATAG
CAAGAGAGGATGAAAATCCAAAGGTTGCTGAAGTTATCTATCCAATAATGC
AGGTTAATGGGATTCATTATCAGGGCGTTGATGTTGCAGTTGGAGGGATGG
AGCAGAGAAAAATACACATGTTAGCAAGGGAGCTTTTACCAAAAAAGGTT

GTTTGTATTCACAACCCTGTCTTAACGGGTTTGGATGGAGAAGGAAAGATG
AGTTCTTCAAAAGGGAATTTTATAGCTGTTGATGACTCTCCAGAAGAGATTA
GGGCTAAGATAAAGAAAGCATACTGCCCAGCTGGAGTTGTTGAAGGAAAT
CCAATAATGGAGATAGCTAAATACTTCCTTGAATATCCTTTAACCATAAAAA
GGCCAGAAAAATTTGGTGGAGATTTGACAGTTAATAGCTATGAGGAGTTAG
AGAGTTTATTTAAAAATAAGGAATTGCATCCAATGCGGTTGAAGAATGCTGT
AGCTGAAGAACTTATAAAGATTTTAGAGCCAATTAGAAAGAGATTA

mCherry-linker18-Leu-eGFP (protein):

MVSKGEEDNMAIIKEFMRFKVHMEGSVNGHEFEIEGEGEGRPYEGTQTAKLK
VTKGGPLPFAWDILSPQFMYGSKAYVKHPADIPDYLKLSFPEGFKWERVMNFE
DGGVVTVTQDSSLQDGEFIYKVKLRGTNFPSDGPVMQKKTMGWEASSERMY
PEDGALKGEIKQRLKLKDGGHYDAEVKTTYKAKKPVQLPGAYNVNIKLDITS
HNEDYTIVEQYERAEGRHSTGGMDELYKHRSQPWVPRARDPPVATLVSKGEE
LFTGVVPILVELDGDVNGHKFSVSGEGEGDATYGKLTLKFICTTGKLPVPWPT
LVTTLTYGVQCFSRYPDHMKQHDFFKSAMPEGYVQERTIFFKDDGNYKTRAE
VKFEGDTLVNRIELKGIDFKEDGNILGHKLEYNYNSHNVYIMADKQKNGIKV
NFKIRHNIEDGSVQLADHYQQNTPIGDGPVLLPDNHYLSTQSALSKDPNEKRD
HMVLLEFVTAAGITLGMDELYKGSGDYKDDDDK

mCherry-linker18-Leu-eGFP (DNA):

ATGGTGAGCAAGGGCGAGGAGGATAACATGGCCATCATCAAGGAGTTCAT
GCGCTTCAAGGTGCACATGGAGGGCTCCGTGAACGGCCACGAGTTCGAGA
TCGAGGGCGAGGGCGAGGGCCGCCCCTACGAGGGCACCCAGACCGCCAA
GCTGAAGGTGACCAAGGGTGGCCCCCTGCCCTTCGCCTGGGACATCCTGT
CCCCTCAGTTCATGTACGGCTCCAAGGCCTACGTGAAGCACCCCGCCGACA
TCCCCGACTACTTGAAGCTGTCCTTCCCCGAGGGCTTCAAGTGGGAGCGCG
TGATGAACTTCGAGGACGGCGGCGTGGTGACCGTGACCCAGGACTCCTCC
CTGCAGGACGGCGAGTTCATCTACAAGGTGAAGCTGCGCGGCACCAACTT
CCCCTCCGACGGCCCCGTAATGCAGAAGAAGACCATGGGCTGGGAGGCCT

CCTCCGAGCGGATGTACCCCGAGGACGGCGCCCTGAAGGGCGAGATCAAG
CAGAGGCTGAAGCTGAAGGACGGCGGCCACTACGACGCTGAGGTCAAGA
CCACCTACAAGGCCAAGAAGCCCGTGCAGCTGCCCGGCGCCTACAACGTC
AACATCAAGTTGGACATCACCTCCCACAACGAGGACTACACCATCGTGGA
ACAGTACGAACGCGCCGAGGGCCGCCACTCCACCGGCGGCATGGACGAGC
TGTACAAGCACCGGTCGCAACCTTGGGTACCGCGGGCCCGGGATCCACCG
GTCGCAACCTTGGTGAGCAAGGGCGAGGAGCTGTTCACCGGGGTGGTGCC
CATCCTGGTCGAGCTGGACGGCGACGTAAACGGCCACAAGTTCAGCGTGT
CCGGCGAGGGCGAGGGCGATGCCACCTACGGCAAGCTGACCCTGAAGTTC
ATCTGCACCACCGGCAAGCTGCCCGTGCCCTGGCCCACCCTCGTGACCACC
CTGACCTACGGCGTGCAGTGCTTCAGCCGCTACCCCGACCACATGAAGCA
GCACGACTTCTTCAAGTCCGCCATGCCCGAAGGCTACGTCCAGGAGCGCA
CCATCTTCTTCAAGGACGACGGCAACTACAAGACCCGCGCCGAGGTGAAG
TTCGAGGGCGACACCCTGGTGAACCGCATCGAGCTGAAGGGCATCGACTT
CAAGGAGGACGGCAACATCCTGGGGCACAAGCTGGAGTACAACTACAACA
GCCACAACGTCTATATCATGGCCGACAAGCAGAAGAACGGCATCAAGGTG
AACTTCAAGATCCGCCACAACATCGAGGACGGCAGCGTGCAGCTCGCCGA
CCACTACCAGCAGAACACCCCCATCGGCGACGGCCCCGTGCTGCTGCCCG
ACAACCACTACCTGAGCACCCAGTCCGCCCTGAGCAAAGACCCCAACGAG
AAGCGCGATCACATGGTCCTGCTGGAGTTCGTGACCGCCGCCGGGATCACT
CTCGGCATGGACGAGCTGTACAAGGGAAGCGGAGACTACAAGGACGACGA
TGACAAG

IFN-α2b-WT (protein):
MCDLPQTHSLGSRRTLMLLAQMRRISLFSCLKDRHDFGFPQEEFGNQFQKAE
TIPVLHEMIQQIFNLFSTKDSSAAWDETLLDKFYTELYQQLNDLEACVIQGVG
VTETPLMKEDSILAVRKYFQRITLYLKEKKYSPCAWEVVRAEIMRSFSLSTNL
QESLRSKEHHHHHH

IFN-α2b-WT (DNA):

ATGTGTGATCTGCCTCAAACCCACAGCCTGGGTAGCCGCCGCACCTTGATG
CTCCTGGCACAGATGCGCCGCATCTCTCTTTTCTCCTGCTTGAAGGACCGC
CATGACTTTGGATTTCCCCAGGAGGAGTTTGGCAACCAGTTCCAAAAGGCT
GAAACCATCCCTGTCCTCCATGAGATGATCCAGCAGATCTTCAATCTCTTCA
GCACAAAGGACTCATCTGCTGCTTGGGATGAGACCCTCCTAGACAAATTCT
ACACTGAACTCTACCAGCAGCTGAATGACCTGGAAGCCTGTGTGATACAG
GGGGTGGGGGTGACAGAGACTCCCCTGATGAAGGAGGACTCCATTCTGGC
TGTGAGGAAATACTTCCAAAGAATCACTCTCTATCTGAAAGAGAAGAAATA
CAGCCCTTGTGCCTGGGAGGTTGTCAGAGCAGAAATCATGAGATCTTTTTC
TTTGTCAACAAACTTGCAAGAAAGTTTAAGAAGTAAGGAACACCATCACC
ATCACCAT

Supplementary Table

Supplementary Table 1: The affinity (*K*D), the association-rate (*k*on) and dissociation-rate (*k*off) of scFv-WT, scFv-CY5, scFv-PEG20K-CY5 and scFv-PEG40K-CY5

	*K*D (M)	*k*on (M^{-1} s^{-1})	*k*off (s^{-1})
scFv-WT	$(1.70 \pm 0.16) \times 10^{-11}$	$(2.03 \pm 0.003) \times 10^{5}$	$(3.45 \pm 0.31) \times 10^{-6}$
scFv-CY5	$(7.21 \pm 0.13) \times 10^{-11}$	$(1.89 \pm 0.003) \times 10^{5}$	$(1.36 \pm 0.02) \times 10^{-5}$
scFv-PEG20K-CY5	$(2.20 \pm 0.05) \times 10^{-10}$	$(6.09 \pm 0.01) \times 10^{4}$	$(1.34 \pm 0.03) \times 10^{-5}$
scFv-PEG40k-CY5	$(4.52 \pm 0.07) \times 10^{-10}$	$(5.45 \pm 0.01) \times 10^{4}$	$(2.46 \pm 0.04) \times 10^{-5}$

Supplementary Table 2: **Primers used in this work**

**Primers**	**Sequences**
pULTRA-pTAFRS-FW	CACAAAGGAGGTGCGGCCGCATGGACGAATTTGAAATGATAAAGAGAAACACATCTG
pULTRA-pTAFRS-RV	CGTTTAAACGCGGCCGCTTATAATCTCTTTCTAATTGGCTCTAAAATCTTTATAAGTTCTTCAGC

pULTRA-R2-74RS-FW	CACAAAGGAGGTGCGGCCGCATGGACAAGAAACCGCTGGA
pULTRA-R2-74RS-RV	CGTTTAAACGCGGCCGCTCACAGGCTGGTGCTAATGCCGTTATAG
pULTRA-vec-FW	GCGGCCGCGTTTAAACGG
pULTRA-vec-RV	GCGGCCGCACCTCCTTTGTG
pCMV-R2-74RS-FW	CCACTCCCAGGTCCAACTGCACGGAAGCTTGCCACCATGGACAAGAAACCGCTGGATGTGC
pCMV-R2-74RS-RV	CAGTCGAGGCTGATCAGCGGGTGGATCCTCACAGGCTGGTGCTAATGCCG
NdeI-pAcFRS-FW	CGCTTTGAGGAATCCCATATGGACGAATTTGAAATGATAAAGAGAAACACATCTG
XhoI-tRNA-RV	CGACCCTGAGCTGCTCGAGC
NheI-FW	GCTAGCGGAGTGTATACTGGCTTACTATG
araBAD-OP-RV	CCTCCTGTTAGCCCAAAAAAACGGG
OP-pAcFRS-FW	CCCGTTTTTTTGGGCTAACAGGAGGAATTAGATCTATGGACGAATTTGAAATGATAAAGAGAAACACATC
salI-pAcFRS-RV	GATGATGATGATGGTCGACTTATAATCTCTTTCTAATTGGCTCTAAAATCTTTATAAGTTCTTCAGC
sfGFP-151TAG-FW	GACACAAACTCGAGTACAACTTTAACTCACACAATGTATAGATCACGGCAGACAAACAAAAGAATGGAATC
sfGFP-151TAG-RV	GATTCCATTCTTTTGTTTGTCTGCCGTGATCTATACATTGTGTGAGTTAAAGTTGTACTCGAGTTTGTGTC
J591Fab-121TAG-FW	GCACCACAGTGACAGTCTCGAGCTAGTCCACCAAGGGCCCATCGG
J591Fab-121TAG-RV	CCGATGGGCCCTTGGTGGACTAGCTCGAGACTGTCACTGTGGTGC
Anti-HER2-scFv-K42TAG-FW	GCATGGTATCAGCAGAAACCAGGGTAGGCCCCTAAGCTCCTGATCTATTCTGC

Anti-HER2-scFv-K42TAG-RV	GCAGAATAGATCAGGAGCTTAGGGGCCTACCCTGGTTTCTGCTGATACCATGC
pcDNA3.1-linker-Leu18TAG-FW	GGGATCCACCGGTCGCAACCTAGGTGAGCAAGGGCGAGGAGCTGT
pcDNA3.1-linker-Leu18TAG-RV	ACAGCTCCTCGCCCTTGCTCACCTAGGTTGCGACCGGTGGATCCC
