# Supplementary material for: Noncanonical amino acids as doubly bio-orthogonal handles for one-pot preparation of protein multiconjugates
Source: Nat Commun. 2023 Feb 21;14:974. doi: 10.1038/s41467-023-36658-y (PMC9944564; doi:10.1038/s41467-023-36658-y)
Supplement: Supplementary file 3 — Reporting Summary [file 41467_2023_36658_MOESM3_ESM.pdf]

nature portfolio

Corresponding author(s): Tao Liu, Xing Yang

Last updated by author(s): Feb 6, 2023

Reporting Summary

Nature Portfolio wishes to improve the reproducibility of the work that we publish. This form provides structure for consistency and transparency in reporting. For further information on Nature Portfolio policies, see our Editorial Policies and the Editorial Policy Checklist.

Statistics

For all statistical analyses, confirm that the following items are present in the figure legend, table legend, main text, or Methods section.

n/a	Confirmed	
☐	☒	The exact sample size (n) for each experimental group/condition, given as a discrete number and unit of measurement
☐	☒	A statement on whether measurements were taken from distinct samples or whether the same sample was measured repeatedly
☐	☒	The statistical test(s) used AND whether they are one- or two-sided<br>*Only common tests should be described solely by name; describe more complex techniques in the Methods section.*
☒	☐	A description of all covariates tested
☒	☐	A description of any assumptions or corrections, such as tests of normality and adjustment for multiple comparisons
☐	☒	A full description of the statistical parameters including central tendency (e.g. means) or other basic estimates (e.g. regression coefficient) AND variation (e.g. standard deviation) or associated estimates of uncertainty (e.g. confidence intervals)
☐	☒	For null hypothesis testing, the test statistic (e.g. F, t, r) with confidence intervals, effect sizes, degrees of freedom and P value noted<br>*Give P values as exact values whenever suitable.*
☒	☐	For Bayesian analysis, information on the choice of priors and Markov chain Monte Carlo settings
☒	☐	For hierarchical and complex designs, identification of the appropriate level for tests and full reporting of outcomes
☒	☐	Estimates of effect sizes (e.g. Cohen's d, Pearson's r), indicating how they were calculated

Our web collection on statistics for biologists contains articles on many of the points above.

Software and code

Policy information about availability of computer code

Data collection	Schrödinger 10.2 was used for pTAF docking with the MjTyrRS crystal structure;<br>Waters Synapt G2-Si (Waters, Manchester, UK) was used for high-resolution mass data collection;<br>Vectra Polaris was used for immunofluorescence of resected specimens;<br>Typhoon FLA 9500 was used for in-gel fluorescence;<br>FluoView1000 confocal microscope was used for confocol images collection;<br>Zeiss LSM880 Confocal microscope was used for confocol images collection;<br>IVIS Spectrum Imaging Systemwas used for in vivo fluorescence imaging(Caliper life Sciences, Hopkinton, MA);<br>PET scanner (Super-Nova, PINGSHENG, Shanghai, China) was used for Micro-PET imaging;<br>The radioactivity was measured using a γ-counter (Packard, Meriden, CT);<br>Octet RED96e instrument (ForteBio Inc., Sartorius, Germany);<br>CytoFLEX flow cytometer (Beckman Coulter, CA, USA).

Data analysis	Living Image 4.3.1 was used for quantification of fluorescence intensity;<br>Avatar software (v1.6.6.5) was used for quantification of Micro-PET intensity;<br>GraphPad Prism v8.0.2 was used for statistical analyses;<br>Octet BLI Analysis 12.2 software was used for the analysis of the binding curves;<br>FlowJo software (Version 10.8.1, FlowJo, Ashland, OR, USA);<br>All data are presented as means ± SEM or means ±SD as depicted in the figure caption. Mann-Whiteny U test was used to compare the differences between two independent samples. Kruskal-Wallis test and Tukey HSD test were used to compare the differences between three or more independent samples. All above statistical analysis was performed with SPSS 26.0. Statistical significance was considered at $P < 0.05$.

For manuscripts utilizing custom algorithms or software that are central to the research but not yet described in published literature, software must be made available to editors and reviewers. We strongly encourage code deposition in a community repository (e.g. GitHub). See the Nature Portfolio guidelines for submitting code & software for further information.

Data

Policy information about availability of data

All manuscripts must include a data availability statement. This statement should provide the following information, where applicable:

- Accession codes, unique identifiers, or web links for publicly available datasets
- A description of any restrictions on data availability
- For clinical datasets or third party data, please ensure that the statement adheres to our policy

The data of High-resolution electrospray-ionization mass, determination of rate constants of all reactions, quantification of fluorescence intensity, biodistribution of 64Cu-labeled antibody fragment in vitro and in vivo, and cytotoxic measurement generated in this study and gels for uncropped versions are provided in the Source Data file. Protein data bank (PDB) files about 1J1U and 6DN0 were obtained from a publicly available source– https://www.rcsb.org. The remaining data generated or analyzed in this study are available within the article and Supplementary Information.

Human research participants

Policy information about studies involving human research participants and Sex and Gender in Research.

Reporting on sex and gender	N/A
Population characteristics	N/A
Recruitment	N/A
Ethics oversight	N/A

Note that full information on the approval of the study protocol must also be provided in the manuscript.

Field-specific reporting

Please select the one below that is the best fit for your research. If you are not sure, read the appropriate sections before making your selection.

☒ Life sciences ☐ Behavioural & social sciences ☐ Ecological, evolutionary & environmental sciences

For a reference copy of the document with all sections, see nature.com/documents/nr-reporting-summary-flat.pdf

Life sciences study design

All studies must disclose on these points even when the disclosure is negative.

Sample size	Sample size was chosen to assure reproducibility of the experiments in accordance with the replacement, reduction and refinement principles of animal ethics regulation. Sample sizes were typically ≥ 3 to ensure sufficent statistics for a proper characterization of the observerd phenomena.
Data exclusions	No data were excluded.
Replication	All attempts at replication were successful. The in vitro data were performed at least twice biologically independently. For the in vivo experiments, ≥ 3 mice per tumor model were used. The detailed description is shown in each figure legend.
Randomization	Throughout the whole experiment, animals were randomized into groups.
Blinding	Blinding was not relevant because this study is not assessing subjective outcomes.

Reporting for specific materials, systems and methods

We require information from authors about some types of materials, experimental systems and methods used in many studies. Here, indicate whether each material, system or method listed is relevant to your study. If you are not sure if a list item applies to your research, read the appropriate section before selecting a response.

Materials & experimental systems

n/a	Involved in the study
☐	☒ Antibodies
☐	☒ Eukaryotic cell lines
☒	☐ Palaeontology and archaeology
☐	☒ Animals and other organisms
☒	☐ Clinical data
☒	☐ Dual use research of concern

Methods

n/a	Involved in the study
☒	☐ ChIP-seq
☐	☒ Flow cytometry
☒	☐ MRI-based neuroimaging

Antibodies

Antibodies used	The primary antibody against PSMA was mouse monoclonal [YPSMA-1] to PSMA (ab19071; abcam), Fitc-labeled goat anti-mouse IgG(H+L) (ZF-0312; ZSGB-Bio) was used for immunofluorescence assay.
Validation	All antibodies were verified by the supplier. All validation statements are found on the respective antibody website. PSMA primary antibody (ab19071) was validated for the following usage: ELISA, WB, IHC-P, IHC-Fr, ICC/IF, Flow Cyt. Fitc-labeled goat anti-mouse IgG(H+L) (ZF-0312) was validated by WB analysis, this antibody reacts with mouse IgG and also with other immunoglobulin light chains of mice, and does not react with non-immunoglobulin serum proteins, but can cross-react with other species of immunoglobulins.

Eukaryotic cell lines

Policy information about cell lines and Sex and Gender in Research

Cell line source(s)	LNCaP (Cat No. SCSP-5021), 22Rv1 (Cat No. SCSP-5022), and PC3 (Cat No. SCSP-532) human prostate cancer cell lines were purchased from the Chinese Academy of Sciences Typical Culture Collection (Shanghai, China). The human breast cancer cell lines HCC1954 (Meisen, China, Cat No. CTCC-003-0205) and MDA-MB-231 (Meisen, China, Cat No. CTCC-001-0019) were obtained as a generous donation from Dr. Yu Cao (Peking University Shenzhen Graduate School). HEK293T cells (Cat No. CRL-11268) were purchased from ATCC.
Authentication	None of the cell lines used were authenticated.
Mycoplasma contamination	All cell lines were tested negative for mycoplasma contamination. Negative status for contamination was verified by MycAwayTM -Color One-Step Mycoplasma Detection Kit from Yeasen.
Commonly misidentified lines (See ICLAC register)	No commonly misidentified cell lines were used in this study.

Animals and other research organisms

Policy information about studies involving animals; ARRIVE guidelines recommended for reporting animal research, and Sex and Gender in Research

Laboratory animals	BALB/c nude mice (4-6 week, male or female) were obtained from the Animal Center at the Peking University Frist Hospital.Mice were group-housed (up to five mice in one cage), maintained at 20–25 °C, 40-60% room humidity and a 12 h light/dark cycle.
Wild animals	This study did not involve wild animals.
Reporting on sex	Prostate cancer only occurs in males, and breast cancer is more common in females.
Field-collected samples	This study did not involve samples collected from the filed.
Ethics oversight	All procedures and protocols were approved by the Animal Ethics Committee at Peking University Frist Hospital (Beijing, China), approval number: J202261. The maximum tumor volume allowed by the Ethics Committee of the Peking University Frist Hospital was 2000 mm3 per tumor, which was not exceeded in these studies.

Note that full information on the approval of the study protocol must also be provided in the manuscript.

Flow Cytometry

Plots

Confirm that:

☒ The axis labels state the marker and fluorochrome used (e.g. CD4-FITC).

☒ The axis scales are clearly visible. Include numbers along axes only for bottom left plot of group (a 'group' is an analysis of identical markers).

☒ All plots are contour plots with outliers or pseudocolor plots.

☒ A numerical value for number of cells or percentage (with statistics) is provided.

Methodology

Sample preparation	Cells were trypsinized, harvested and washed with PBS and then analysed with flow cytometer. In some experiments, cells were stained with antibodies or probes according to the manufacturer's protocols, and then analyzed by flow cytometry.
Instrument	CytoFlex Flow Cytometer (Beckman Coulter, CA, USA)
Software	FlowJo software (Version 10.8.1, FlowJo, Ashland, OR, USA).
Cell population abundance	During sample measurements and initial gate was used to ensure a cell count of 10,000 cells or events was collected of a relevant cell population.
Gating strategy	Cell populations were gated for a live population using FSC-A and SSC-A plot of cell only sample. in order to remove cell aggregates and small debris, followed by single-cell gate (SSC-A and SSC-H). This live population was then used in fluorescent histograms.

☒ Tick this box to confirm that a figure exemplifying the gating strategy is provided in the Supplementary Information.
